# cCPE Fusion Proteins as Molecular Probes to Detect Claudins and Tight Junction Dysregulation in Gastrointestinal Cell Lines, Tissue Explants and Patient-Derived Organoids

**DOI:** 10.3390/pharmaceutics15071980

**Published:** 2023-07-19

**Authors:** Ayk Waldow, Laura-Sophie Beier, Janine Arndt, Simon Schallenberg, Claudia Vollbrecht, Philip Bischoff, Martí Farrera-Sal, Florian N. Loch, Christian Bojarski, Michael Schumann, Lars Winkler, Carsten Kamphues, Lukas Ehlen, Jörg Piontek

**Affiliations:** 1 Clinical Physiology/Nutritional Medicine, Medical Department, Division of Gastroenterology, Infectiology, Rheumatology, Charité—Universitätsmedizin Berlin, 12203 Berlin, Germany; ayk.waldow@charite.de (A.W.); lbeier@bwh.harvard.edu (L.-S.B.); 2 Laboratory of Mucosal Barrier Pathobiology, Department of Pathology, Brigham and Women’s Hospital, Harvard Medical School, Boston, MA 02115, USA; 3 Berlin Institute of Health (BIH), Charité—Universitätsmedizin Berlin, BIH Center for Regenerative Therapies (BCRT), 13353 Berlin, Germany; 4 Department of Anesthesiology and Intensive Care Medicine, Charité—Universitätsmedizin Berlin, 10117 Berlin, Germany; 5 Charité—Universitätsmedizin Berlin, Corporate Member of Freie Universität Berlin and Berlin Institute of Health, Institute of Pathology, 10117 Berlin, Germany; 6 Berlin Institute of Health, Charité—Universitätsmedizin Berlin, 10178 Berlin, Germany; 7 German Cancer Consortium (DKTK), Partner Site Berlin, and German Cancer Research Center (DKFZ), 69120 Heidelberg, Germany; 8 Department of General and Visceral Surgery, Campus Benjamin Franklin, Charité—Universitätsmedizin Berlin, 12203 Berlin, Germany; 9 Medical Department, Division of Gastroenterology, Infectiology, Rheumatology, Charité—Universitätsmedizin Berlin, 12203 Berlin, Germany; 10 Experimental Pharmacology & Oncology Berlin-Buch GmbH, 13125 Berlin, Germany; 11 Park-Klinik Weißensee, Department of General-Visceral and Minimally-Invasive Surgery, 13086 Berlin, Germany

**Keywords:** claudin, tight junction, clostridium perfringens enterotoxin, colon, adenoma, tumor, gastric dysplasia, gastric cancer, organoids

## Abstract

Claudins regulate paracellular permeability, contribute to epithelial polarization and are dysregulated during inflammation and carcinogenesis. Variants of the claudin-binding domain of *Clostridium perfringens* enterotoxin (cCPE) are highly sensitive protein ligands for generic detection of a broad spectrum of claudins. Here, we investigated the preferential binding of YFP- or GST-cCPE fusion proteins to non-junctional claudin molecules. Plate reader assays, flow cytometry and microscopy were used to assess the binding of YFP- or GST-cCPE to non-junctional claudins in multiple in vitro and ex vivo models of human and rat gastrointestinal epithelia and to monitor formation of a tight junction barrier. Furthermore, YFP-cCPE was used to probe expression, polar localization and dysregulation of claudins in patient-derived organoids generated from gastric dysplasia and gastric cancer. Live-cell imaging and immunocytochemistry revealed cell polarity and presence of tight junctions in glandular organoids (originating from intestinal-type gastric cancer and gastric dysplasia) and, in contrast, a disrupted diffusion barrier for granular organoids (originating from discohesive tumor areas). In sum, we report the use of cCPE fusion proteins as molecular probes to specifically and efficiently detect claudin expression, localization and tight junction dysregulation in cell lines, tissue explants and patient-derived organoids of the gastrointestinal tract.

## 1. Introduction

The gastrointestinal mucosa lines the digestive system and forms the barrier between the environment and the internal milieu. While it enables the absorption of nutrients and water during digestion, it must rigorously exclude the passage of undesirable luminal contents, such as microorganisms and toxins, to submucosal compartments [1]. With few exceptions, epithelial cells lining the alimentary canal are circumferentially attached to each other by the apical junction complex, comprising tight junctions (TJs), adherens junctions and desmosomes. These cell–cell contacts facilitate the formation of a continuous physical barrier by sealing the paracellular space and provide tensile strength by integrating the cytoplasmic actin, myosin and cytokeratin networks [2,3]. TJs, the apicalmost constituent of the apical junction complex, seal the intercellular space against uncontrolled diffusion of water and solutes. Here, the intercellular space is eliminated and the outer leaflets of apposing plasma membranes seem to fuse [4]. Proteins of the claudin family (CLDNx) [5], which are encoded by ~27 genes in mammals [6,7,8], form the characteristic TJ strands that are observed in freeze–fracture electron microscopy [9], and determine the tightness and size- and charge-selective permeability of the epithelium [1,5,8,10]. Throughout the gastrointestinal tract, expression patterns of claudins vary depending on the permeability demands of the epithelium (stomach: CLDN1 to −4, −7, −18.2, −23; small intestine: Cldn1 to −5, −7, −8, −10, −12, −15, −23; colon: 1, −3, −4, −5, −7, −8, 12, −15, −23 [11,12]).

Changes in claudin expression patterns and claudin mislocalization are associated with numerous gastrointestinal diseases. In inflammatory bowel diseases, disturbances in the regulation of water and solute transport and macromolecule uptake are observed [11]. These changes are regularly associated with increased expression of CLDN2, which forms a paracellular sodium and water channel and contributes to barrier loss [13,14,15,16]. Delocalized claudins might be related to permeability increases, which have been described as a risk factor for the development and clinical relapse of active Crohn’s disease [17,18]. Loss of cell–cell adhesion is a landmark of tumor progression. While junctional claudins support cell polarity, non-junctional claudins have been described to be involved in the migration, invasion and metastasis of cancer cells [19,20,21]. Studies on claudin expression in various cancers provide heterogeneous data, though upregulation of CLDN1, −3 and −4 has been repeatedly reported for colon cancer, and up- and dysregulation of CLDN4 and −18.2 have been shown in gastric tumors [22,23,24,25]. Nonetheless, alteration in TJ barrier integrity through claudin dysfunction in gastric and other gastrointestinal cancers remains to be investigated in detail [26].

Altogether, during inflammation and tumorigenesis, TJ impairment is associated with a shift from junctional and mainly polymerized [8,27] to extra-junctionally located and primarily unpolymerized claudins [28,29,30]. Alteration of the polymerization state of claudins in inflammatory diseases, cancer and other pathological conditions has been indicated by electron microscopy, STED microscopy, FRET assays and other methods [15,27,30,31,32]. Thus, disease-associated, mislocalized claudins present important diagnostic markers. The extracellular segments (ECSs) of claudins provide molecular targets on the cell surface that are accessible in non-junctional claudins and become mostly inaccessible upon polymerization into TJ strands [31,33,34,35]. Hence, the state of the gastrointestinal epithelium can be gauged by the number of accessible claudins on the surface of the epithelial cells. Fluorescence endoscopy with highly specific molecular probes could conceivably supplement white light endoscopy during routine screenings to implement the detection of delocalized claudins. This could aid in predicting the onset of active inflammation or in detection of (pre-)cancerous lesions and guide appropriate therapies.

The ECSs of a subset of claudins, mainly CLDN3 and −4 in the gastrointestinal tract, serve as receptors for the C-terminal, claudin-binding domain of Clostridium perfringens enterotoxin (cCPE) [33,35,36,37,38]. Polymerization of claudins into TJ strands renders the cCPE binding site inaccessible [8,31,33]. The prominent formation of complexes of ligand cCPE and receptor claudins, which represent an aberrant cellular state, e.g., inflammation or carcinogenesis, can be exploited to specifically detect overexpressed and/or mislocalized claudins in a disease context [31].

To foster the implementation of cCPE as a claudin-targeting tool for diverse applications, claudin-binding properties of cCPE have been altered by structure-based mutagenesis [37,39,40,41,42,43]. The variant cCPE-S305P/S307R/S313H (cCPE-SSS) binds with high affinity to a broad spectrum of claudins (CLDN1 −9, −14, −19) [39,42,44,45], including relevant claudins that are expressed in the gastrointestinal tract and frequently deregulated during disease. This renders cCPE-SSS a promising and highly sensitive probe to detect disease-specific claudin mislocalization and overexpression. The mutant cCPE-Y306A/L315A (cCPE-YL) does not exhibit any claudin binding and serves as negative control [39,46]. Applicability of differently tagged cCPE-based tools for barrier opening or claudin detection by immunocytochemistry, live-cell imaging or flow cytometry has been demonstrated previously [37,39,40,44,45,47,48,49,50]. However, for instance, the random and multiple conjugations of amino-reactive fluorophores compromised stability and claudin-binding properties to a certain extent.

To study disease- and tissue-specific traits of novel probes, adequate experimental models are needed. Tissue- or patient-derived explants are a versatile system for ex vivo investigation. However, propagation under laboratory conditions is limited, restricting their use to one-time experiments [51]. Patient-derived organoids are 3D multicellular structures that are generated from surgically resected tissue specimens or biopsies. They preserve the identity of the parental tissue and can be propagated in vitro to be implemented as living biobanks, thus enabling repetition and standardization of experimental setups [52]. Therefore, patient-derived organoids are excellent models for investigating the applicability of molecular probes in a tissue- and patient-specific disease context [53].

The aim of this work was to study and verify the potential of cCPE-based molecular probes to detect deregulated claudins under pathological conditions. We established a yellow fluorescent protein (YFP)-cCPE probe and analyzed ex vivo cCPE binding to deregulated claudins in colonic tissue explants in the context of inflammation and in early neoplastic stages. Furthermore, we generated patient-derived organoids from gastric dysplasia and gastric cancer, characterized them and demonstrated that claudins expressed in the parental tissue can be specifically detected in organoids by YFP-cCPE probes.

## 2. Materials and Methods

The compositions of culture media and buffers, which are not explicitly stated in this section, can be found in the supplement (Tables S1 and S2). Compounds, additional cell lines, plasmids, primers, equipment, and software are listed in Table S3. Antibodies and their respective dilution, manufacturer and species can be found in Table S5 of the supplement. Additional methodological details can be found in Table S4 and Methods S1–S7 in the supplement. Details on manufacturers and catalog numbers are also contained in the supplement (Tables S1, S3 and S5).

### 2.1. Protein Preparations

Methods for plasmid generation, protein purification and fluorophore-labeling are described in detail in the Supplementary Methods (Methods S1–S3).

### 2.2. Cell Culture

HT-29/B6 (human colonic epithelial cell line previously established at Clinical Physiology/Nutritional Medicine) [31,54], HEK293 (human embryonic kidney cell line [41,55], Sigma-Aldrich, Taufkirchen, Germany), HEK293 cells stably transfected with p3xFLAG-CMV-10/huCLDN4 (HEK293-CLDN4) described previously [55] and MDCK-C7 (dog kidney epithelial cell line [56], gift from Hans Oberleithner, Department of Physiology, University of Münster, Germany) were grown as adherent cultures in sterile flasks and used for a maximum of 10 passages after thawing. HT-29/B6 cells were cultured and grown at high confluency for 12–14 days before they were plated for experiments as described previously [31,54]. HEK293 and MDCK-C7 cells were grown to confluency for 4 to 5 days before experiments, and culture media were changed three times a week. Culture media compositions are listed in Table S1 in the supplement.

### 2.3. Plate Reader Binding Assay

cCPE binding assays in a plate reader format were performed similarly to those described previously [39] using YFP fluorescence as readout. For details, see Supplementary Method S4.

### 2.4. Ex vivo Incubation of Tissue Specimens with GST-cCPE

Preparation and glutathione S-transferase (GST)-cCPE incubation of a rat colon and ex vivo cCPE treatment of human colon polyps are described under Supplementary Methods S5 and S6, respectively.

### 2.5. Generation and Characterization of Patient-Derived Gastric Cancer Organoids

#### 2.5.1. Gastric Tissue Processing

Tissue specimens were mechanically extracted from patients with histologically confirmed gastric cancer who underwent gastrectomy at the Department of General and Visceral Surgery, Campus Benjamin Franklin, Charité—Universitätsmedizin Berlin, Germany. Tissue specimens of gastric tumors and adjacent macroscopically unsuspicious healthy gastric tissue were collected in tubes containing a cooled washing medium (see Table S2). The average time from surgical removal of the primary tissue to initiation of tissue dissociation was 30 min. In instances where samples could not be processed immediately, a modified protocol for cryogenic preservation was implemented. Briefly, tissue was cut into 3 mm fragments, fetal bovine serum with 10% DMSO was added and the tissue was slowly cooled to −80 °C. The tumors of the originating specimen were examined by pathologists of the Institute of Pathology, Charité—Universitätsmedizin Berlin. Healthy tissue samples also underwent histological examination. Procedures were performed on ice and under sterile conditions using a laminar flow hood. Upon arrival at the laboratory, the tissue was rinsed in 3 mL washing medium. A part of the tissue was fixed in a 4% formaldehyde solution for 24 h for subsequent embedding in paraffin.

#### 2.5.2. Gastric Organoid Culture

The largest part of the tumor specimen to be used for cell culture was weighed, covered in 0.5 mL washing medium (see Table S1) in a 35 mm Petri dish, and mechanically dissociated into small fragments using a sterile blade and forceps. Enzymatic digestion was performed with a freshly prepared enzyme mix (see Table S1). Half of the enzyme mix to be added was placed on the mechanically dissociated tissue and mixed with a cut pipette tip. The tissue was digested for 30 min at 37 °C. Next, the tissue medium mixture was rinsed over the Petri dish several times with a cut pipette tip, chopped with a sterile blade and treated with a syringe stamp. In the second enzymatic digestion step, the remaining half of the enzyme mix was added, and the samples were incubated for 45 min at 37 °C with horizontal rotation at 75 rpm. After enzymatic digestion, samples were transferred to a 100 µm cell strainer placed on a 50 mL tube. One mL of washing medium was rinsed over the cell strainer and Petri dish repeatedly to incorporate the maximum amount of the digested tissue. Cells in the flow-through were counted using a counting chamber and were centrifuged at 300–440× *g* for 5 min at 25 °C. The cell pellet was resuspended in a mix of organoid medium (see Table S1, adapted from [52,57]) and 75% Matrigel.

The conditioned medium used for the organoid medium was derived from L-WRN cells as described previously [58]. Seeding onto Matrigel-coated cell culture plates and solidification at 37 °C for 30 min were performed before primary cell culture medium including 5% Matrigel was added. Cells were cultured at 37 °C in a humidified incubator with 5% CO_2_. The medium was changed every three to four days, and cell growth and organoid formation were observed daily under the light microscope. The organoids were detached when they comprised more than 75% of the volume of the cell culture dish. For this purpose, the medium was carefully removed, 0.5 mL of 4 °C cold Dulbecco’s Phosphate Buffered Saline (DPBS) or TrypLE Express was added, and organoids within the Matrigel were incorporated using a 1000 µL pipette tip and were placed in a 15 mL tube with 8 mL of cold (4 °C) DPBS. The cell culture dish was rinsed again with DPBS, and the solution was added to the 15 mL tube. The suspension was centrifuged at 500× *g* at 4 °C for 5 min, and the supernatant was removed until the surface of the Matrigel cell suspension at the bottom of the tube was reached. The cells were resuspended in a mix of organoid medium and Matrigel or Geltrex and placed in two wells of a Matrigel-coated cell culture dish as described previously [59]. Resuspension of the cell pellet in 100% Matrigel or Geltrex, seeding in domes (50 µL in 24 wells), solidification at 37 °C for 30 min and subsequent addition of organoid medium were performed.

### 2.6. Live-Cell Imaging

#### 2.6.1. Live-Cell Imaging of HT-29/B6 with AF647-GST-cCPE and YFP-cCPE

For live-cell imaging, HT-29/B6 cells were plated onto cover glasses (⌀ 32 mm) at 60% confluency. After 24 or 48 h, culture media were removed and replaced by media containing 10 µg/mL cCPE GST-cCPE, Alexa Fluor647-conjugated GST-cCPE or YFP-cCPE). Cover glasses were mounted to fit the heated microscope stage (37 °C, 5% CO_2_), and culture media were substituted by HEPES Ringer solution to facilitate pH stability, supplemented with 10 µg/mL of the respective cCPE variant and CellMask Deep Red plasma membrane stain (1:15,000). Image acquisition with an LSM780 started minutes after the media were replaced, and signals were stable after 30 min.

#### 2.6.2. Live-Cell Imaging of Colonic Ulcers

Human transverse colon biopsies showing colonic ulcers were obtained during routine endoscopy. Tissue biopsies were immediately placed in 0.9% (*w*/*v*) NaCl solution on ice and processed within 30 min.

Biopsies were transferred to coverslips in a heating chamber mounted on the stage of an LSM780 incubated at 37 °C with 5% CO_2_ atmosphere. The solution was replaced by 111-Ringer solution with substrate supplemented with 15 µg/mL of YFP-cCPE-SSS or –YL and Hoechst 33342 (1:2500). Live-cell imaging was performed for up to 120 min; after 20 min, strong YFP signals were already obtained.

#### 2.6.3. Live-Cell Imaging of Gastric Organoids

For incubation of living gastric organoids cultured in 8-well glass-bottom Lab-Tek II chamber slides, the culture media were replaced by primary cell culture medium containing 30 µg/mL of YFP-cCPE-SSS or YFP-cCPE-YALA (negative control) and CellMask deep red plasma membrane stain (1:15,000). During image acquisition, cells were kept at 37 °C in a 5% CO_2_ atmosphere. Imaging was performed with an LSM780 using an LD C-Apochromat 40×/1.1 water immersion objective. After 120 min, permeation of the organoid by YFP-cCPE-SSS stagnated. Then, the culture medium was removed, and the organoids were washed with DPBS and fixed with 4% PFA and 0.5% glutaraldehyde. Histogel embedding and deparaffinization were conducted as described in Method S7.4.

### 2.7. Histology and Immunochemistry

Histology and immunochemistry procedures are reported in detail in Supplementary Method S7.

### 2.8. Quantification of cCPE Binding in Whole Mount Samples

Z-stacks of tissue samples were acquired using a Zeiss laser scanning microscope LSM780. The binding of unconjugated GST-cCPE was detected by mouse-anti-GST and AlexaFluor594-conjugated anti-mouse antibodies. The binding of AlexaFluor647-conjugated GST-cCPE was directly detected by the respective fluorescence. Maximum intensity projections of z-stacks were created with Zen Black. cCPE binding was calculated as (mean intensity in cCPE channel)/(mean intensity in DAPI channel) × (relative area of image with cCPE signal intensity above background threshold). Statistical significance was determined by using the non-parametric Kruskal–Wallis test with a post hoc Dunn test.

### 2.9. Flow Cytometry

#### 2.9.1. Flow Cytometry for Assessing YFP-cCPE Binding

Confluent cells were harvested by trypsinization (0.25% Trypsin, 2.21 mM EDTA), 5 min for HEK293 and HEK293-CLDN4, 20 min for MDCK-C7 at 37 °C, followed by gentle scraping, quenching of trypsin with excess medium and centrifugation (300× *g*, 4 °C). Subsequently, cells were washed three times with cold PBS (-Ca^2+^/-Mg^2+^), followed by incubation with LIVE/DEAD Fixable Violet Dead Cell Stain (1:1000) for 10 min on ice. Following one washing step with PBS (-Ca^2+^/-Mg^2+^), cells were resuspended in ice-cold flow buffer and incubated with YFP-cCPE fusion protein, ChromoTek GFP-Booster ATTO647N (1:500) and CellMask Orange plasma membrane stain (1:1000) (10 µg/mL each) (30 min, 4 °C, dark). Finally, they were washed twice with flow buffer and stored on ice in the dark until analysis. Right before flow cytometry measurement, cell suspensions were passed through a cell strainer into Falcon 5 mL round-bottom polystyrene tubes.

Flow cytometry measurements were conducted manually on a BD LSRFortessa X-20. Fluorescence compensation was applied using samples stained with only one of the respective stains. Unstained samples for each cell line were used to account for autofluorescence and noise. Intensities were measured for forward scatter (FSC), sideward scatter (SSC) and fluorescence intensities at 405 nm, 488 nm, 594 nm and 647 nm. Data acquisition was performed in BD FACSDiva, while data analysis was performed at Floreada.io. YFP and ATTO647N fluorescence were measured. For clarity, only the YFP channel is shown here.

#### 2.9.2. Flow Cytometry of Gastric Organoids

Gastric organoids were dissociated with TrypLE Express until a single-cell suspension was observed. Cells were counted using a C-chip (Neubauer improved DHC-N01); 1 × 10^6^ cells were transferred to a V-bottom plate and centrifuged at 500× *g* and 4 °C for 5 min. The supernatant was removed, and a staining mix containing 1:200 anti-PDGFRα, 1:200 anti-CD44, 1:400 anti-CD166, 1:400 anti-CD45, 1:100 anti-EpCAM, 1:400 anti-CD31 and 1:400 anti-CD133 antibodies as well as 1:400 Zombie Aqua Fixable Viability Kit and 1:50 Fc block was added and incubated at 4 °C for 15 min. Then, 150 µL FACS buffer was added, followed by centrifugation at 400× *g* and 4 °C for 5 min. The supernatant was aspirated, and the sample was resuspended again in 100 µL FACS buffer. Flow cytometry was carried out using a CytoFlex, and analysis was performed using FlowJo software (Version 10.8.2).

### 2.10. Targeted DNA Sequencing

DNA from fresh frozen tumor tissue and organoid cells was extracted with Quick-DNA Miniprep Plus Kit according to the manufacturer’s instructions. DNA purity and concentration were examined using a spectrophotometer and a fluorometer, respectively. Targeted next-generation sequencing (NGS) was performed using the multiplex PCR-based Ion AmpliSeq Cancer Hotspot panel v2 on the Ion GeneStudio S5. Each library contained eight primer pairs for determining a specific sample ID according to the single-nucleotide polymorphism (SNP) signature.

## 3. Results

### 3.1. YFP-cCPE-wt and YFP-cCPE-SSS Bind Specifically to CLDN4 and Other Claudins on the Cell Surface

**Figure 1:**
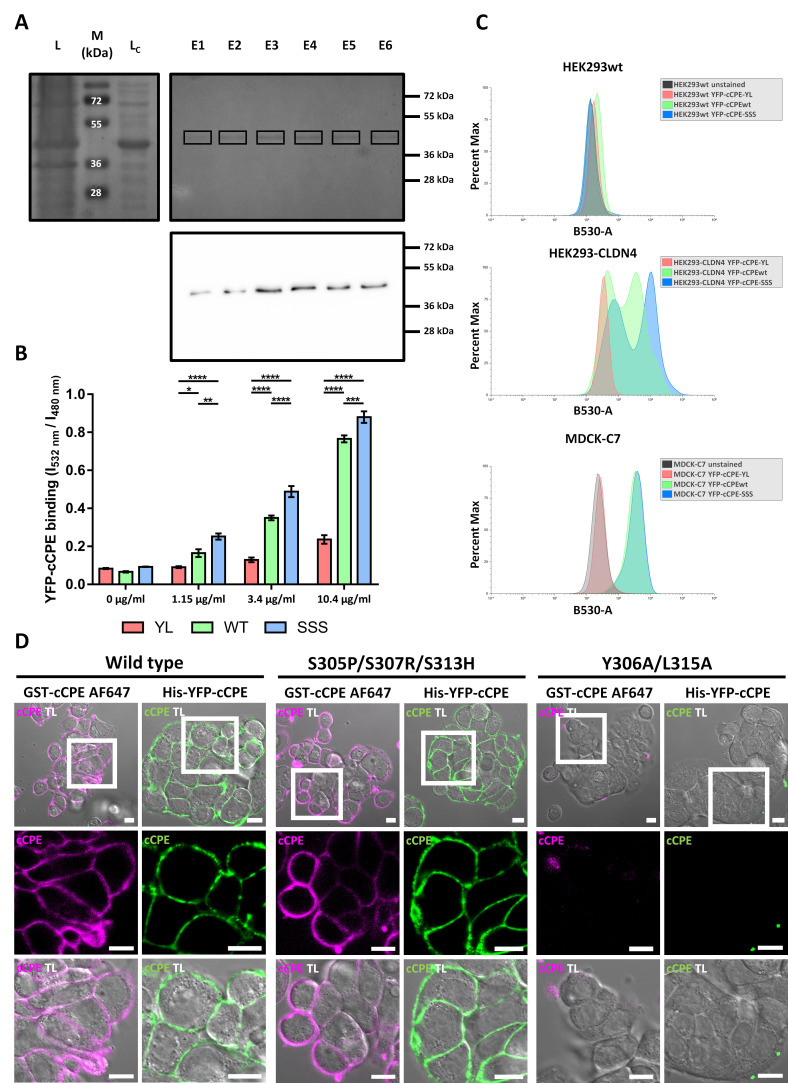
YFP-cCPE fusion proteins bind to claudins on the cell surface. (**A**) Purification of YFP-cCPE-wt. SDS PAGE of *E. coli* lysate (L), cleared lysate (L_C_) and eluate fractions (E1-6). Upper, Coomassie Blue-stained gel showing ~40 kDa band (squares) corresponding to YFP-cCPE-wt being enriched in the eluate fractions. Lower, Western blot of eluate fractions showing a ~40 kDa band detected by anti-GFP/YFP antibody, identifying the purified recombinant protein as full-length YFP- cCPE-wt. (**B**) Cellular plate reader binding assay. Binding of YFP-cCPE fusion proteins to CLDN4-transfected HEK293 cells. Concentration-dependent binding observed for YFP-cCPE-wt and YFP-cCPE-SSS, but not YFP-cCPE-YL. Mean + SEM, n = 4. Two-way ANOVA with post hoc Tukey test to account for multiple comparisons. Comparisons indicated in graph; *p* > 0.05, * *p* < 0.05, ** *p* < 0.01, *** *p* < 0.001, **** *p* < 0.0001. (**C**) Exemplary flow cytometry experiment with YFP-cCPE-labeled HEK293, HEK293-CLDN4 and MDCK-C7 cells. YFP-cCPE-SSS and -wt exhibit claudin-specific binding in HEK293-CLDN4 and MDCK-C7. (**D**) Sub-confluent HT-29/B6 cells after incubation with fluorescent cCPE-wt, -SSS and -YL. Insets show close-up regions of interest at cell–cell contacts. Claudins are ubiquitously detected on the cell surface with claudin-binding cCPE variants, but not with cCPE-YL, confirming claudin-specific binding by YFP-cCPE-wt and -SSS. Fluorescence and transmission channels are shown. TL, transmitted light channel. Scale bars, 10 µm.

Previously, we used GST-cCPE fusion proteins as claudin binders and claudin-specific TJ modulators. cCPE-wt and different mutants thereof were used to target different claudin subtypes [33,37,39,40,44,45]. To enable direct detection of cCPE fusion proteins, for instance for live-cell imaging, YFP-cCPE fusion proteins were generated by subcloning, expression in *E. coli* and his-tag-based purification (see Section 2 and Figure 1A). In total, three different YFP-cCPE variants were generated: YFP-cCPE-wt, binding preferentially to CLDN3 and −4; the broad-spectrum binder YFP-cCPE-S305P/S307R/S313H (YFP-cCPE-SSS); and the non-binding control, YFP-cCPE-Y306A/L315A (YFP-cCPE-YL).

First, the YFP-cCPE fusion proteins were tested using HEK293 cells that do not express endogenous claudins and HEK293 cells transfected with CLDN4 (HEK293-CLDN4). The binding of YFP-cCPE was assessed using a plate reader assay [39] with YFP fluorescence as the readout. YFP-cCPE-wt and YFP-cCPE-SSS bound in a concentration-dependent manner to HEK293-CLDN4 cells with maximal binding at 10.4 µg/mL. The mutation S305P/S307R/S313H enhanced the binding slightly over YFP-cCPE-wt, while the negative control YFP-cCPE-YL showed no or negligible binding (Figure 1B). Thus, YFP-cCPE-wt and YFP-cCPE-SSS bound specifically in a claudin-dependent manner to CLDN4 on the surface of living HEK293-CLDN4 cells, similar to what was shown for the corresponding GST-cCPE fusion proteins [45].

We further confirmed the specific detection of claudins by YFP-cCPE using flow cytometry (Figure 1C). HEK293wt, HEK293-CLDN4 and MDCK-C7 were each incubated with the different YFP-cCPE fusion proteins (wt, -SSS, -YL). No YFP fluorescence was detected for claudin-free HEK293wt cells incubated with any YFP-cCPE variants (Figure 1C, upper panel). The HEK293-CLDN4 cell line showed two major subpopulations upon YFP-cCPE-wt and –SSS incubation, represented by two pronounced peaks in the respective histograms. A highly YFP-positive subpopulation and a low-YFP-positivity subpopulation were present, indicated by increased YFP signal in comparison to the baseline level of the negative control YFP-cCPE-YL. The different subpopulations detected are likely due to the polyclonal properties of the used HEK293-CLDN4 cell line. In line with the plate reader assay, flow cytometry confirms a slightly higher affinity of YFP-cCPE-SSS over YFP-cCPE-wt towards CLDN4 (Figure 1C, middle panel).

MDCK-C7 cells served as a standard in vitro model for epithelial cells expressing a multitude of endogenous claudins (CLDN1, −3, −4, −5, −7 and −8, [60]) and forming TJs. YFP fluorescence was detected for MDCK-C7 cells incubated with YFP-cCPE-wt or YFP-cCPE-SSS but not for untreated or YFP-cCPE-YL-incubated cells. However, none of the claudin-binding YFP-cCPE variants showed an increased binding over the other in MDCK-C7 cells (Figure 1C, lower panel). The flow cytometry data demonstrate that YFP-cCPE fusion proteins can be used for specific, direct and quantitative detection of individual living cells having different levels of claudins on their surface.

Lastly, we confirmed the claudin-specific binding of YFP-cCPE fusion protein by live-cell imaging of HT-29/B6 cells, an established in vitro model for colonocytes [31]. Incompletely differentiated HT-29/B6 cells were used to minimize claudin polymerization into TJ strands, which presumably masks the extracellular domain of claudins and thus hinders cCPE binding [8,31,35]. HT-29/B6 cells were incubated with Alexa Fluor647-conjugated GST-cCPE fusion proteins (AF647-GST-cCPE) or YFP-cCPE fusion proteins. In the case of AF647-GST-cCPE, cCPE-K257A was used as a wt-like binder, since this mutation prevents fluorophore coupling close to the claudin-ECS2 binding pocket of cCPE without interfering with claudin binding [37,39]. YFP-cCPE-wt and –SSS bound to the endogenously expressed claudins and were detected on the surface of the colonocytes—similar to AF647-GST-cCPE fusion proteins. The lack of cCPE-YL binding demonstrated the specificity of the binding (Figure 1A,D).

In sum, the data demonstrate that YFP-cCPE fusion proteins can be used for specific and direct detection of claudins on the surface of living cells.

### 3.2. cCPE Fusion Proteins Visualize Non-Junctional Claudins and TJ Diffusion Barriers in Colonocyte Islands

**Figure 2:**
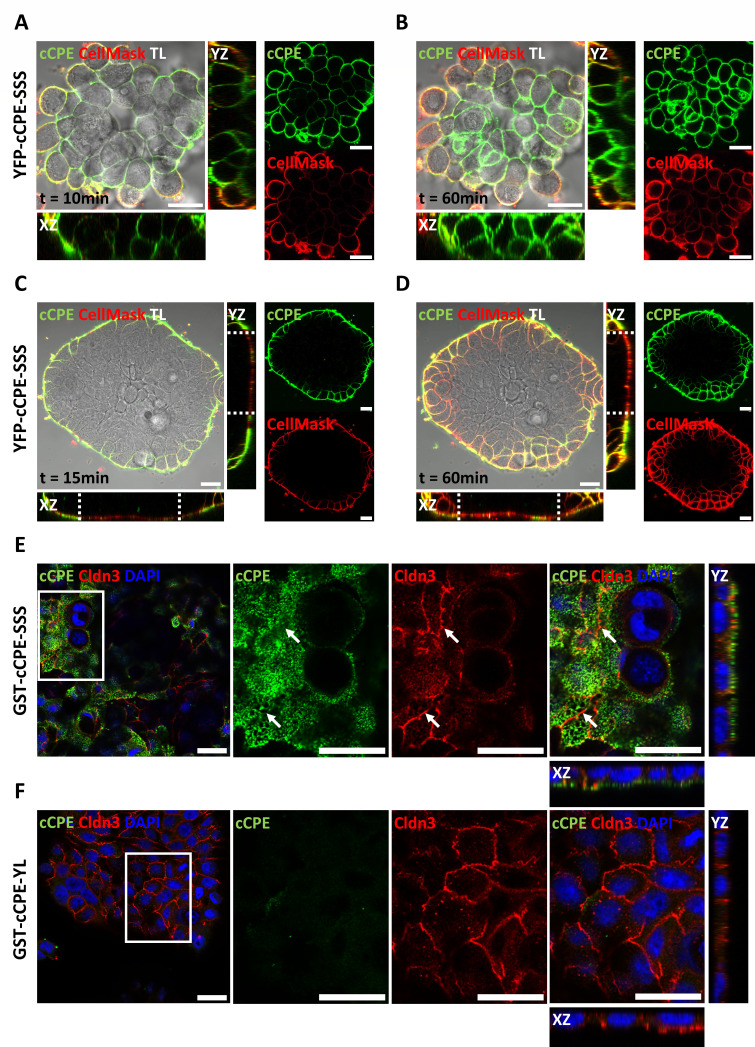
cCPE fusion proteins bind extra-junctional claudins on HT-29/B6 cells. (**A**–**D**) Live-cell imaging of YFP-cCPE-SSS binding to and permeation of sub-confluent HT-29/B6 cell islets. Incubation with cCPE and the membrane marker CellMask for the indicated time at two varying stages: (**A**,**B**) one day after plating, small islets with weakly differentiated cells; (**C**,**D**) two days after plating, larger islets with differentiated cells at the center. TL, transmitted light channel. (**A**,**C**) At both stages, colonocyte islets showed strong peripheral YFP-cCPE-SSS and CellMask staining within 15 min incubation time. After this short incubation period, two-day-old HT-29/B6 islets showed an apical exclusion and active diffusion barrier within the center of the islet, confined by the dotted line in the xz/yz orthogonal view of the confocal z-stack (**C**). (**B**) After 60 min, the small, one-day-old islets showed complete permeation by YFP-cCPE-SSS, confirming the absence of a TJ barrier. (**C**) In contrast, the larger, two-day-old islets prevent the free diffusion of molecules through the cell layer. (**C**,**D**) Z-stacks of two-day-old islets show exclusion of YFP-cCPE-SSS and CellMask in central parts of colonocyte islets confined by the dotted lines, while peripheral cells of islets do not hinder either marker and show strong YFP-cCPE-SSS and CellMask signal. (**E**,**F**) HT-29/B6 grown in high cell density for two days, incubated with GST-cCPE-SSS or -YL, fixed and stained against GST-cCPE (anti-GST), CLDN3 and nuclei (DAPI). Close-ups (indicated by white squares) show orthogonal view of z-stacks, revealing exclusive detection of apical extra-junctional claudins by cCPE in a punctual fashion. Arrows indicate areas with poor colocalization between junctional CLDN3 and GST-cCPE-SSS. GST-cCPE-YL showed no specific signal. Scale bars, 20 µm.

Development of TJ barriers in HT-29/B6-cell layers was monitored by live-cell imaging of cell islets at different time points after plating and YFP-cCPE-SSS application (Figure 2A–D). CellMask was applied as a membrane-binding tracer. One day after cell plating, YFP-cCPE-SSS bound to peripheral cells within 10 min and homogeneously to the cell surface of all cells of the islets within 60 min, indicating full accessibility of claudins (Figure 2A,B). In contrast, two days after plating, central cells of colonocyte islets exhibited YFP-cCPE-SSS and CellMask exclusion in the lateral and basal membrane, even after 60 min of incubation. Nevertheless, the central apical membrane was strongly stained by CellMask and showed only weak punctuate signals for YFP-cCPE-SSS, corresponding to non-junctional claudins (Figure 2C,D). This indicates the formation of a TJ diffusion barrier and inaccessibility of claudins in the TJs and the underlying basolateral membranes of non-peripheral cells within the cell group. In contrast, the peripheral cells showed an abundant YFP-cCPE-SSS and CellMask signal at apical and basolateral membranes, which gradually permeated peripheral cell membranes over time, hence representing the lack of a TJ diffusion barrier (Figure 2C,D). The results demonstrated that YFP-cCPE fusion proteins can be efficiently used for imaging of non-junctional claudins in the plasma membrane of living cells and detection of paracellular diffusion barriers.

To substantiate that cCPE binds, at least preferentially, to non-junctional claudins, HT-29/B6 cells were grown at a higher cell density to promote TJ formation. Immunocytochemistry of fixed cells showed that apically applied GST-cCPE-wt bound strongly to the apical membrane but not to the apicolaterally localized TJ detected by CLDN3 accumulation. In line with YFP-cCPE-SSS incubation, GST-cCPE-wt showed punctuate apical signals and low spatial overlap with the continuous apicolateral CLDN3 signal (Figure 2E). In addition, cCPE did not reach the basolateral membrane, or only did to a very small extent, indicating the formation of a TJ diffusion barrier (Figure 2E). GST-cCPE-YL did not bind to the cells, verifying the claudin dependence of the cCPE binding (Figure 2F). The results showed that YFP-cCPE fusion proteins are a reliable tool for the visualization of non-junctional claudins in the plasma membrane of living cells and the detection of paracellular diffusion barriers.

### 3.3. Use of cCPE Variants for Detection of Delocalized Non-Junctional Claudins in Ex Vivo Models of Rat and Human Colon Tissue

**Figure 3:**
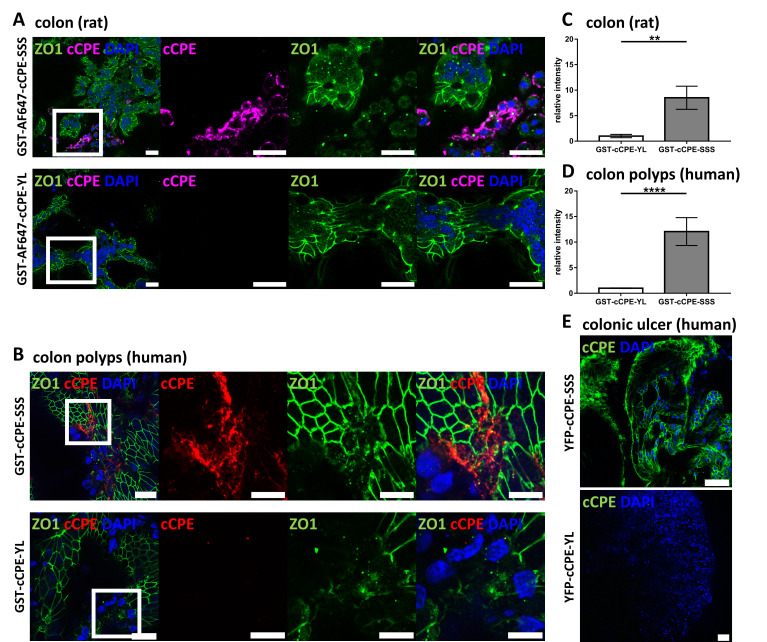
Claudin detection via cCPE fusion proteins in ex vivo colon models. (**A**) Rat colon dissected and mounted in Ussing chambers incubated with AF647-GST-cCPE-SSS and –YL. Close-ups (indicated by white squares) show cCPE-positive areas with punctuate discontinuous ZO1 signal, indicating dysfunctional TJ barrier and non-junctional claudins. Scale bar = 20 µm. (**B**) Human colon polyps (adenoma) from biopsies incubated with GST-cCPE-SSS and –YL, detected via secondary anti-GST antibody. Close-ups (indicated by white squares) show areas with punctuate ZO1 signal permeated by GST-cCPE-SSS, indicating disturbed TJ barrier. Scale bar = 20 µm. (**C**,**D**) Quantification of cCPE relative to DAPI signal in maximum intensity projections. Relative cCPE intensity normalized to cCPE-YL intensity. GST-cCPE-SSS shows considerably higher relative intensity over GST-cCPE-YL, indicating strong binding of GST-cCPE-SSS to epithelial tissue with impaired TJs in both rat colon and human colon polyps. Mean ± SEM, Kruskal–Wallis test with post hoc Dunn test: ** *p* = 0.003; n = 2 for cCPE-SSS, n = 1 for cCPE-YL with 5 individual images per sample quantified for (**C**) and n = 5 for (**D**) with 10 individual images analyzed per cCPE incubation. Mann–Whitney test: **** *p* < 0.0001. (**E**) Live imaging of whole mount human transverse colon biopsy showing colonic ulcer with YFP-cCPE-SSS and -YL. YFP-cCPE-SSS detects claudins on cell surface, while YFP-cCPE-YL does not show signal. Scale bars, 20 µm.

To test whether non-junctional claudins in primary tissue can be detected by cCPE, an ex vivo model of rat colon tissue was used. Colon mucosa was prepared from a rat and cultured in Ussing chambers, and the barrier integrity was monitored by TER measurements [61]. The tissue was incubated with AF647-GST-cCPE fusion proteins for 30 min and subsequently fixed, followed by immunostaining and confocal microscopy for image acquisition. cCPE-SSS binding was detected only in a subpopulation of cells, where epithelial areas showed non-continuous and punctuate ZO-1 signals, indicative of compromised TJs. Epithelial areas with a continuous, TJ-like ZO-1 signal showed no cCPE-SSS binding, indicating a functional paracellular barrier. Furthermore, for the negative control cCPE-YL, almost no signals were obtained in any areas of the colon mucosa (Figure 3A). Quantification of cCPE signals yielded an approximately 8 times higher signal of cCPE-SSS (mean = 8.51 ± 2.26) than cCPE-YL (mean = 1.0 ± 0.33) (Figure 3C).

The nature of the cCPE-positive areas with fragmented TJs in the rat colon tissue was not further analyzed in the course of this study. However, cCPE-positive areas with deranged TJs have been previously identified in fully differentiated and barrier-forming HT-29/B6 cell cultures (on filter inserts). They might be the result of continuous physiological junctional rearrangements in the epithelia or local TJ dysregulation due to impairing factors [31].

TJ dysregulation is assumed to be part of epithelial dedifferentiation and carcinogenesis. Thus, we investigated biopsies of human colorectal adenomas. Similar to what was detected for the rat colon tissue, cCPE-SSS binding was detected in epithelial areas with fragmented, non-continuous and punctuate ZO-1 signals, indicating compromised TJs. In contrast, no clear cCPE-SSS signal was detected in epithelial areas with a continuous, TJ-like ZO-1 signal (Figure 3B). Specificity and claudin dependence of the cCPE signals was verified by comparing cCPE-SSS and cCPE-YL incubations. In line with the results from the rat colon, quantification of cCPE-SSS (mean = 12.06 ± 2.73) in human colorectal adenomas yielded an approximately 12 times higher signal over cCPE-YL (mean = 1.00 ± 0.05) (Figure 3D).

Additionally, TJ dysregulation occurs also during inflammation [62]; therefore, cCPE-based claudin detection was tested in biopsies of inflamed human colon tissue. Endoscopically resected biopsies of a colonic ulcer were directly imaged live using confocal microscopy. YFP-cCPE-SSS gave a strong basolateral signal in epithelial cells, unlike YFP-cCPE-YL, showing that claudins can be detected in human inflamed colon tissue by YFP-cCPE-based live imaging. However, TJ integrity could not be assessed due to YFP-cCPE-SSS being applied from both luminal and basal sides (Figure 3E).

In sum, the data obtained with the cell lines, the different tissue samples and differently tagged and labeled cCPE fusion proteins showed that cCPE variants can specifically and efficiently be used for the detection of non-junctional and potentially dysregulated claudins in different in vitro and ex vivo models of the gastrointestinal epithelium under physiological and pathological conditions. 

### 3.4. Use of YFP-cCPE to Probe the Expression, Polar Localization and Dysregulation of Claudins in Human Gastric Cancer Organoids

#### 3.4.1. Establishment of Patient-Derived Gastric Organoids as a 3D Testing Platform for Cancer-Related Dedifferentiation

**Figure 4:**
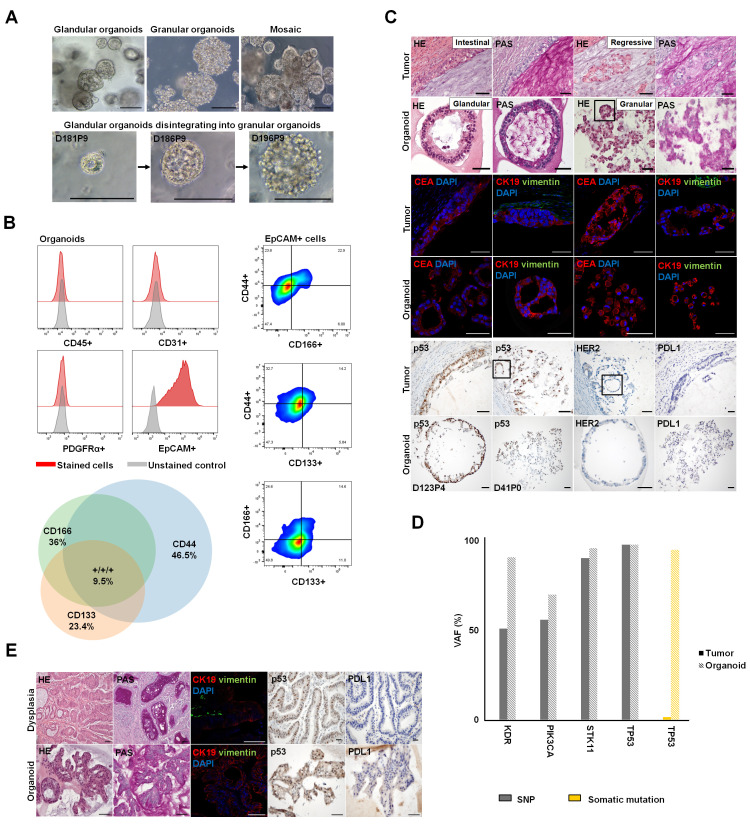
Generation of patient-derived organoids from gastric tumor and gastric dysplasia. (**A**) Successful establishment of GC_1 gastric cancer organoid line, expanded with stable growth for >7 months. The culture had a mosaic morphology of glandular and granular, discohesive organoids. Organoids with mixed morphology and glandular organoids changing their phenotype by disintegration to granular organoids could be observed. D: days in culture; P: passage. (**B**) Left: Flow cytometry analysis showed that organoids comprise EpCAM+ epithelial cells. No immune cells (CD45^+^), endothelial cells (CD31^+^) or fibroblasts (PDFGRα^+^) were present in the organoid culture. Right: Cell populations with distinct expression patterns of the stem cell markers CD133, CD44 and CD166 were enriched in the Epcam^+^ cells. Lower: Venn diagram showing fractions of stem cell subsets with single, double or triple CD44+, CD133+, CD166+ phenotype. (**C**) The organoid line preserved the histological properties of the parental tumor. HE/PAS staining: Tumor cells and organoids showed pleomorphic, hyperchromatic nuclei, eosinophilic cytoplasm and small amounts of PAS-positive mucin. IHC: CEA and CK19 positivity were shown. Vimentin-positive tumor stroma and vimentin-negative, epithelial-only organoids could be observed. The parental tumor cells as well as glandular and granular organoids were negative for PDL1 and HER2. The primary tumor tissue had heterogeneous p53 expression. Organoids had p53-negative cells and p53-positive cells with strong nuclear p53 localization. The fraction of cells with strong nuclear p53 expression increased in culture. Squares: Representative glandular formations within regressive tumor cell formations and granular/discohesive organoids. Scale bars, 50 µm. (**D**) Targeted DNA sequencing of 51 cancer-related genes in the organoid line and parental tumor. Known SNPs in KDR Exon 11 (c.1416 A > T, p.Q472H), PIK3CA Exon 7 (c.1173 A > G, p.I391M), STK11 Intron 3 (c.465-51 T > C) and TP53 Exon 4 (c.215 C > G, p.P72R) and cancer driver mutation in TP53 Exon 7 (c.715_720del, p.N239_S240del) were detected in organoid cells in correspondence with the primary tumor. For the cancer driver mutation in TP53 Exon 7 (p.N239_S240del), variant allele frequency (VAF) was much higher for the organoid line. (**E**) Establishment of glandular organoids from GC_2 high-grade dysplasia. Parental tissue and organoids comprised dysplastic cells with enlarged hyperchromatic nuclei and few intracellular PAS-positive cells. The cell signal of CK19 in glandular organoids was similar to that observed in the corresponding high-grade dysplasia. Vimentin-positive cells could be observed in the parental tumor stroma but not in epithelial-only organoids. Primary tissue and organoids shared p53 wild-type status with p53-positive and -negative cells and negativity for PDL1. Scale bars, 50 µm.

We established patient-derived organoids from a gastric adenocarcinoma (intestinal type according to Lauren’s criteria) that underwent neoadjuvant therapy and from a high-grade gastric dysplasia as a proof-of-concept model to test cancer-related epithelial dedifferentiation. Here, we focused on the use of YFP-cCPE to probe the expression, polar localization and pathophysiological dysregulation of claudins. The gastric adenocarcinoma (GC_1) presented a mosaic growth pattern of either small glands or discohesive single carcinoma cells as a morphological indicator for tumor regression after neoadjuvant therapy (Table 1, Figure 4A). This mosaic phenotype was reflected in the organoid line via glandular organoids as well as discohesive, granular organoids (Figure 4A). Organoids could be cryopreserved, thawed and expanded for over 7 months and multiple passages. Flow cytometry verified that organoids comprise EpCAM+ epithelial cells. No immune cells, endothelial cells or fibroblasts were present in the organoid culture. The stem cell markers CD133, CD44 and CD166 were enriched in EpCAM+ cells (Figure 4B). To verify that organoids preserve the identity of their parental tissue, comparative analyses were performed. Histological facets of the primary tissues were maintained in the organoid line. The parental tumor cells showed slightly enlarged, pleomorphic and hyperchromatic nuclei of variable size and shape without visible nucleoli. The cytoplasm was eosinophilic and contained small amounts of PAS-positive mucin. Organoids demonstrated a similar morphology; however, some of the cells had a higher nucleus-to-cytoplasm ratio. Immunohistochemically, the parental tumor cells revealed a cytoplasmic positivity for CEA and CK19. Organoids showed similar expression patterns with additional membranous CEA expression. The cells of the tumor stroma stained positive for the mesenchymal cell marker vimentin, whereas the tumor cells and epithelial-only organoids were vimentin-negative. Negativity for PDL1 and HER2, decisive cancer-associated biomarkers, was shared between the parental tumor and organoids. Furthermore, tumor and organoid cells showed a heterogeneous nuclear expression of the tumor suppressor protein p53, indicative of a preserved *TP53* wild-type status. However, the fraction of p53-positive cells increased in the organoid culture, indicative of an enrichment of *TP53* mutated tumor cell subclones (Figure 4C). We performed targeted DNA sequencing of 51 cancer-related genes. Known SNPs in *KDR* Exon 11 (c.1416 A > T, p.Q472H), *PIK3CA* Exon 7 (c.1173 A > G, p.I391M), *STK11* Intron 3 (c.465-51 T > C) and *TP53* Exon 4 (c.215 C > G, p.P72R) and a cancer driver mutation in *TP53* Exon 7 (c.715_720del, p.N239 _S240del) were detected in organoid cells in correspondence with the primary tumor. The somatic *TP53* p.N239_S240del mutation was detected with a variant allele frequency (VAF) of 1.5% in the primary tumor with a tumor cell content of <10%. VAF increased to 95% in the organoid line, indicating a selective enrichment of tumor cells in our organoid culture (Figure 4D).

Further, organoids from GC_2 were established as a model to assess claudin dysregulation in high-grade dysplasia (Table 1). While routine histopathological analysis showed a mucinous adenocarcinoma, the primary tissue that was used for organoid generation comprised high-grade dysplasia. The generated glandular organoids conserved histological features of the parental tissue. Organoids with hyperchromatic nuclei reflected the parental tissue with dysplastic cells. The dysplastic epithelial cells in parenteral tissue as well as the epithelial-only organoids were negative for vimentin. Both organoids and GC_2 gastric dysplasia showed a p53 wild-type pattern, CK19 positivity and PDL1 negativity (Figure 4E).

#### 3.4.2. Characterization of Epithelial Differentiation in Gastric Tissue

**Figure 5:**
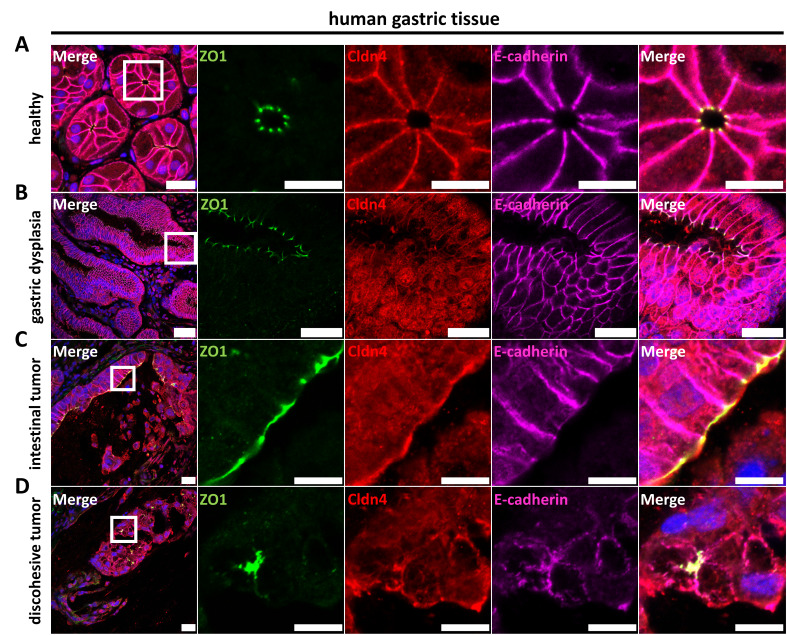
Immunocytochemistry of paraffin-embedded sections of primary human gastric tissue. (**A**) Healthy gastric tissue showed CLDN4 in the lateral membrane, apicolateral ZO1 and lateral E-cadherin. CLDN4 strongly colocalized with E-cadherin in the lateral membrane and apicolaterally with ZO1. (**B**) High-grade gastric dysplasia (GC_2) showed CLDN4 in intracellular compartments and at lateral cell–cell contacts, apicolateral ZO1 and lateral E-cadherin, indicative of proper polarization and TJ formation. (**C**) GC_1 tumor islets with intestinal differentiation showed apicolateral ZO1 and basolateral E-cadherin expression, indicative of epithelial polarity. CLDN4 showed varying enrichment at apical membranes and partially colocalized with ZO1, but did not appear in the lateral membrane and hence did not colocalize with E-cadherin. (**D**) Discohesive tumor cells from GC_1 with regressive alteration displayed no clear polarization with diffuse ZO1, diffuse CLDN4 and non-polar E-cadherin expression. Discohesive tumors showed no indication of proper TJ formation. White squares indicate close-ups shown to the right. Scale bars, 50 µm; close-ups, 10 µm.

Claudins, CLDN4 in particular, have been associated with gastric tumorigenesis [22,24]. However, mechanisms of crosstalk between claudins, other TJ proteins and gastric cancer and the morphological representations remain unclear. Thus, we investigated cellular polarity and junctional protein localization in healthy gastric tissue, GC_2 high-grade dysplasia and a GC_1 gastric tumor by immunohistochemistry of paraffin-embedded sections. For the healthy gastric tissue, proper cell polarity and junctional integrity were evidenced by localization of the nuclei to the basal side of the cell, restriction of E-cadherin and CLDN4 to the basolateral cell membrane and restriction of ZO1 to the apicolateral TJ domain where it colocalized with CLDN4. In addition, gland-like tissue architecture was prominent (Figure 5A). High-grade gastric dysplasia also showed CLDN4 expression at lateral cell-contacts, apicolateral ZO1 expression and lateral E-cadherin expression. Due to morphology and junctional marker distribution, the epithelium in gastric dysplasia appears to maintain proper polarization and TJ formation (Figure 5B).

The GC_1 gastric tumor with intestinal differentiation and regions with discohesive cells showed a variation in the expression of ZO1, E-cadherin and CLDN4: Although the overall tissue organization/architecture differed for the tumor regions with intestinal differentiation with respect to the healthy gastric glands, formation of epithelial monolayers facing luminal structures and cell polarity was still detectable to a large extent. While E-cadherin was restricted to the abluminal, basolateral side, ZO-1 was localized to the apicolateral membrane domain where it at least partly colocalized with CLDN4 (Figure 5C). In contrast, for tumor regions with discohesive cells, altered after neoadjuvant therapy, no clear formation of epithelial monolayers facing luminal structures was detected. In addition, neither ZO1, CLDN4 nor E-cadherin showed a polar distribution (Figure 5D). Thus, tissue organization, cell polarity and presumably TJ barrier formation were strongly disturbed for tumor regions with discohesive cells.

#### 3.4.3. Claudin Expression, as Well as Differential Cell Polarity and Junctional Integrity, is Largely Conserved between Parental Tissues and Organoids

YFP-cCPE incubation was used to probe the expression and presence of (cCPE-receptor) claudins on the surface of the living organoids. For glandular and granular organoids from gastric tumor GC_1, YFP-cCPE-SSS but not the YFP-cCPE-YL negative control showed strong fluorescence (Figure S1). This showed that both organoid types expressed (cCPE-receptor) claudins, similar to what was detected for the respective parental tumors. However, the distribution of YFP-cCPE differed drastically between the organoid types.

**Figure 6:**
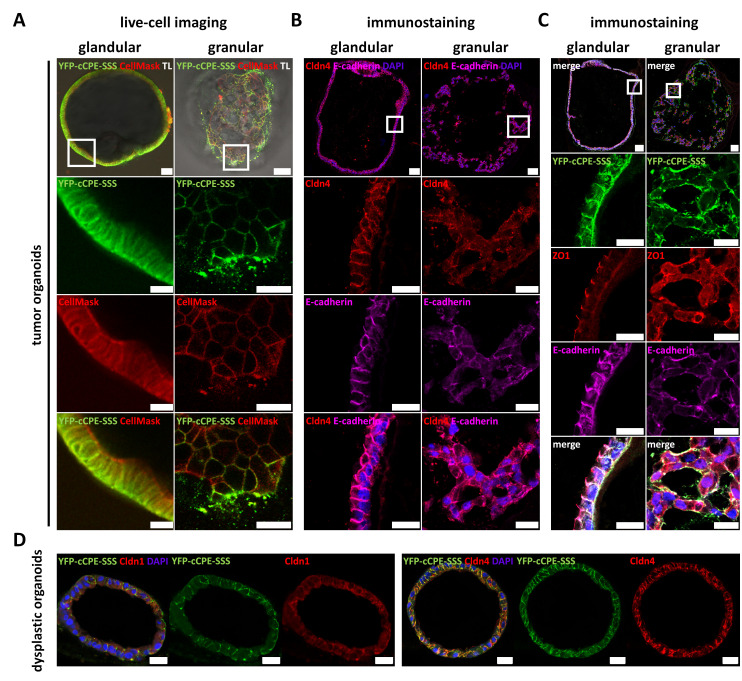
Mosaic gastric organoid line shows differential junctional marker expression in live-cell imaging and immunofluorescence. (**A**) Live-cell imaging with basolaterally applied YFP-cCPE-SSS and CellMask of glandular (left) and granular (right) organoids from GC_1. Close-ups show representative sections of the whole organoids. Glandular organoids showed only basolateral YFP-cCPE-SSS staining, while CellMask showed basolateral and partial apical staining. This indicated polarization of cells in organoids capable of lumen formation and a TJ barrier, which is permeable for small membrane-binding molecules but impermeable for large molecules such as YFP-cCPE and/or claudins. Granular organoids showed no apparent polarization and no clear lumen or barrier formation, since CellMask and YFP-cCPE-SSS permeated the whole organoid. TL, transmitted light channel. Scale bar, 20 µm; close-ups, 10 µm. (**B**,**C**) Immunostaining of fixed samples. Organoids, embedded in histogel and paraffin and fixed in 4% PFA, stained for CLDN4, E-cadherin, YFP and ZO1. Glandular and granular organoids showed the same labeling pattern for YFP-cCPE-SSS as in live-cell imaging. In short, glandular organoids showed lateral/apicolateral CLDN4, lateral E-cadherin, apicolateral ZO1 and basolateral YFP staining, highly indicative of intact TJs and proper polarization. Granular organoids, however, showed diffuse CLDN4, diffuse E-cadherin, diffuse ZO1 and diffuse YFP signals, highly indicative of non-polarized cells, which lack TJs. Scale bars, 20 µm; close-ups, 10 µm. (**D**) Paraffin-embedded and PFA-fixed organoids generated from GC_2 representing dysplastic organoids. Organoids showed a glandular morphology. They were incubated with YFP-cCPE-SSS prior to fixation, followed by subsequent staining of YFP, CLDN4 and CLDN1. YFP-cCPE-SSS and CLDN4 showed mostly lateral signals, while the CLDN1 signal was diffuse. Overall dysplastic organoids appeared polarized with lumen and TJ formation similar to tissue. White squares indicate close-ups shown below. Scale bars, 20 µm.

For the glandular type, YFP-cCPE-SSS labeled the surface cell layer continuously surrounding the largely cell-free organoid lumen and was restricted to the abluminal, basolateral membrane. In contrast, in the granular-type organoids, cell surfaces within the aggregate were ubiquitously labeled by YFP-cCPE-SSS, and the signal was not confined to a specific domain of the cell membranes (Figure 6A). This indicated cell polarity and the formation of a TJ diffusion barrier for the glandular but not the granular organoids.

This was further analyzed by immunocytochemistry of paraffin-embedded sections of YFP-cCPE-incubated organoids. For the glandular type, YFP-cCPE-SSS was restricted to the basolateral membrane. In particular, YFP-cCPE-SSS colocalized with CLDN4 and E-cadherin in the lateral membrane. ZO-1 was restricted to the apicolateral TJ domain and was present at each cell–cell contact, indicating the formation of a continuous TJ belt completely surrounding the organoid lumen. YFP-cCPE and E-cadherin signals reached close to the ZO1 signal, but in contrast to the CLDN4 signal, they did not colocalize with ZO-1, indicating that YFP-cCPE and E-cadherin did not cross the apicolateral ZO-1-positive domain, further supporting the presence of functional TJs and cell polarity (Figure 6B,C).

Also, for the high-grade dysplasia GC_2, immunocytochemistry of paraffin-embedded sections of YFP-cCPE-incubated organoids showed glandular morphology and colocalization of YFP-cCPE-SSS with CLDN4 in the lateral membrane. For comparison, with CLDN1, also expressed in the healthy stomach and in gastric cancer, no clear and discrete colocalization was observed (Figure 6D).

In sum, YFP-cCPE fusion proteins were used to analyze the TJ phenotype in patient-derived organoids as a 3D in vitro model for gastric dysplasia and gastric cancer via broad-spectrum claudin detection. The presence of functional TJs and cell polarity in glandular organoids and, in contrast, a disrupted diffusion barrier in granular organoids was revealed. In future studies, YFP-cCPE-based probing of the TJ phenotype in such a patient-derived organoid system could be used for either mechanistic analysis or testing of potential targets for anticancer therapies.

## 4. Discussion

The high-affinity interaction between cCPE and a subset of members of the claudin protein family—the CPE receptor claudins (CLDN3, −4, −6 to −9, −14, −19)—is well established [33,35,36,37,38,39,46,66]. Mutations in cCPE have been used to expand, narrow or shift the claudin subtype preference [37,39,40,41,42,44,45]. In general, all of these cCPE variants bind to more than one claudin subtype. In contrast, several antibodies were described to be specific for a particular claudin subtype (e.g., CLDN1, −4, −5 or −18.2) [67,68,69,70]. For most applications, narrow specificity is desirable, for instance, to reduce potential side effects of anti-CLDN18.2 antibodies that are in clinical trials to treat gastric or gastroesophageal junction cancer [71,72,73,74]. However, in some cases, it is advantageous to address multiple claudins by cCPE variants in order to increase the number of targeted protein molecules and thus the efficacy of target-mediated effects, and to avoid target claudins escaping from a single specific claudin antibody [44]. Further, YFP-cCPE can be used for the generic sorting of cells expressing any of the cCPE-receptor claudins. This could be employed, for instance, to sort claudin-high versus claudin-low tumor organoid cells for drug screening or mechanistic analysis. In addition, GST-cCPE or YFP-cCPE fusion proteins can be used for the pull-down assay of complexes formed by multiple claudins and associated proteins [27,37,44,75]. From an economic viewpoint, recombinant cCPE protein can be expressed and purified more resource- and cost-effectively (E. coli) than antibodies. In addition, since the structure and mechanisms of the cCPE–claudin interaction are known, cCPE claudin-binding properties can be shifted or blocked by site-directed mutagenesis. This includes the generation of variants that lack only the ability for claudin binding. Such a mutant (cCPE-YL) was used in this study to exclude claudin-independent, non-specific binding of the cCPE fusion proteins. In addition to this control and cCPE-wt, and to target more claudins, we used cCPE-SSS as a variant with broadened claudin-subtype binding properties (high-affinity binding at least also to CLDN1, −2, −5) [39,42,44]. However, in this study, we only exemplarily included it as a cCPE variant, but we did not quantitatively compare the binding (extent, subcellular pattern) of cCPE-wt and –SSS to endogenously expressed claudins. This can be performed in more detailed studies in the future.

Another advantage of cCPE fusion proteins is that they can be expanded in a modular manner: (i) with other toxin domains, e.g., for cytotoxic targeting of claudin-overexpressing tumors (full-length CPE variants or cCPE-PSIF fusions) [41,48,76], (ii) to generate xenon biosensors for magnetic resonance imaging [55] or radiolabeled probe-based SPECT imaging allowing detection of claudin-overexpressing cells and tumors [77], (iii) to engineer functionalized nanoparticles to target prostate adenocarcinoma in in vitro and in vivo models [78,79], or (iv) fluorophore conjugation [80,81] or—as in this study—fluorescent protein fusion for fluorescence-based detection of claudin-expressing cancer cells in vitro, ex vivo and in vivo. Thus, cCPE fusion proteins are flexible and useful additions to the diverse toolbox of claudin binders.

Similar to antibodies, cCPE fusion proteins can sequester or trap claudins, sterically inhibiting their renewal in TJs and thus over time opening paracellular barriers [40,44,47,82]. However, if this effect is unwanted, as in this study, it can be prevented by reducing the concentration and incubation time of cCPE. It was shown that long-term incubation (>4 h) with high concentrations of cCPE promotes tight junction disassembly and barrier opening, whereas short-term incubation (<1 h) and low concentrations do not impair epithelial barriers [37,39,40,44,45,47,48,49,50].

Here, we used GST- and YFP-cCPE variants for simultaneous microscopic detection of the multiple cCPE variant-binding claudins expressed in the cells of interest (HT29-B6: CLDN1 to −5, −7, −8 [83]; colon tissue: CLDN1, −3 to −5, −7, −8 [11]; gastric tissue and organoids CLDN1 to −4, −6, −7 [12,74], this study).

While showing similar claudin-binding properties to GST-cCPE fusion proteins, YFP-cCPE enabled direct use for live-cell imaging. We showed that YFP-cCPE binds in a claudin-dependent manner and substantiated the preferential binding of cCPE to non-junctional claudins on the cell surface of gastrointestinal epithelial cells. cCPE binding to claudins outside of TJs was indicated previously [31,33,34,35,47] but also questioned [38]. However, due to steric interference, direct binding of cCPE to intact claudin polymers within TJs is not compatible with current structural tight junction strand models [8,27,84,85,86]. However, differences in size (for instance, 6xhis-cCPE ~15 kDa versus GST-cCPE/YFP-cCPE ~42 kDa/~80 kDa for monomer/dimers) of tagged cCPE might influence the accessibility of claudins in close proximity to the junctional claudin strands. However, the extent to which small cCPE variants are able to bind to claudin extracellular domains that become locally accessible due to partial claudin dissociation within TJ strand meshworks has to be investigated. Nevertheless, in this study, GST- and YFP-cCPE clearly bound prominently to non-junctional regions, but did not bind or hardly bound to the apicolateral TJ region, characterized by strong enrichment of CLDN3 or ZO1 (Figure 2, Figure 3 and Figure 6C).

In sum, YFP-cCPE-based probing of non-junctional claudins in living and fixed cells provides information about the functional state of TJs, such as the shift between non-junctional pools in the apical and/or basolateral membrane and junctional pools in the apicolateral membrane. In addition, it can be used to trace the paracellular permeability for large molecules, and thus TJ barrier integrity. Further, with YFP-cCPE, cell polarity and polar distribution of its receptor claudins can be assessed.

Pathogenic non- and missense mutations have been described for many claudins including CLDN1 (NISCH syndrome), −2 (obstructive azoospermia), −5 (alternating hemiplegia), −10 (HELIX syndrome), −14 (DFNB29 deafness), −16 and −19 (both FHHNC syndrome) [1,27,86]. Although not explicitly investigated, based on the cCPE-binding consensus motif [35,44], none of these mutations are predicted to considerably affect the cCPE binding properties of the respective claudins directly. Thus, under a given pathologic condition, cCPE binding is expected to depend mainly on the expression level and non-junctional cell surface localization of CPE receptor claudins rather than on claudin mutations.

As a proof of concept, we used cCPE-based claudin detection in different in vitro and ex vivo models differing in species background and TJ integrity. We demonstrated that this detection strategy can be used for a broad range of samples and questions of interest. Exemplarily, we intensified the use of YFP-cCPE for claudin detection and TJ phenotyping of gastric dysplasia and gastric cancer organoid models. To this end, we established a protocol for the generation of gastric cancer organoids. The organoids generated reflected the properties of the primary tumor tissue and showed two different morphological phenotypes (glandular and granular appearance). Using YFP-cCPE, we demonstrated that both organoid populations share cell surface expression of cCPE receptor claudins (including CLDN4) but differ in cell polarization and TJ barrier integrity. Importantly, this difference was detected for living organoids.

In future studies, this cCPE-based phenotyping could be used for further claudin detection as well as characterization and sorting of different organoid populations of different epithelial dedifferentiation status, different patients or after different treatment protocols. For instance, it could be tested whether a certain cytotoxic treatment affects different organoid populations (cCPE-/claudin-positive vs. cCPE-/claudin-negative, with intact TJ vs. with compromised TJ) to a differing extent. For this, it will be important to compare organoids derived from healthy and cancer tissue, for instance, with respect to glandular vs. granular morphology, claudin expression and TJ integrity.

CLDN18.2 is strongly expressed in the stomach but not in other tissues and is dysregulated in gastric cancer, and CLDN18.2-antibody-mediated targeting has been tested in clinical trials for cancer treatment [73,74]. However, this non-classic claudin is not recognized by any cCPE variant [35]. Thus, it would be of interest to compare the cell surface presence of CLDN18.2 (via antibodies) and (via YFP-cCPE) that of cCPE-receptor claudins indicated to be expressed in gastric cancer (CLDN1 to −4, −6, −7 [12,74]) in parental tumor tissue as well as the respective organoids. This might improve the knowledge about the mechanistic contribution of the different claudins to gastric cancer progression and their potential as prognostic markers and targets for treatment.

## 5. Conclusions

We show that functional YFP-cCPE proteins can be designed, expressed and purified with low effort. YFP fusion overcomes the need for chemical fluorophore conjugation bearing the risk of reducing claudin binding and protein stability.

We further report the use of cCPE variant fusion proteins as molecular probes for the generic detection of multiple claudins regarding their expression, localization and tight junction dysregulation in living or fixed samples of gastrointestinal cell lines, tissue explants and gastric organoids. In sum, our study provides evidence that YFP-cCPE can be used as a mechanistic and diagnostic tool to assess claudin dysregulation in gastrointestinal disease and other conditions.

## Figures and Tables

**Table 1:** Characteristics of patient donors. Histomorphology as assessed in clinical routine and by pathologists for the purpose of this study. AC = adenocarcinoma. Extension of disease described via TNM classification of the “International Union Against Cancer”. Grading was assessed. Tumor subtype according to Lauren classification or WHO classification 2019 [63,64]. Tumor regression grading: 1 a: no residual tumor, complete regression; 1 b: <10% residual tumor, subtotal regression; 2: 10–50% residual tumor, partial tumor regression; 3: >50% residual tumor, minimal to no tumor regression [65].

Patient	Histology	Subtype	Morphology	T	N	M	G	R	L	V	Neoadjuvant Therapy	Tumor Regression
GC_1	AC	Intestinal (Lauren)	Glandular, regressive changes	3	1	1	-	0	0	0	Yes	1b
GC_2	AC + high grade dysplasia	Mucinous (WHO 2019)	Mucinous	1b	0	0	3	0	0	0	No	-

## Data Availability

Data are contained within the article or the above-mentioned supplementary material, and data are available on request due to privacy, ethical or other restrictions.

## Supplementary Materials

The following supporting information can be downloaded at: https://www.mdpi.com/article/10.3390/pharmaceutics15071980/s1 [87,88,89,90,91,92], Figure S1: Live-cell imaging of gastric cancer organoids with YFP-cCPE-SSS and –YL; Tables S1: Culture media for cell lines and gastric organoid culture; Table S2: Solution and buffers; Table S3: Reagents and resources; Table S4: Immunofluorescence staining protocol for HT-29/B6 cells and whole mount samples of rat colon and colon polyp biopsies; Table S5: Primary and secondary antibodies and fluorophores; Method S1: Plasmids; Method S2: Purification of 6×His-eYFP-cCPE proteins; Method S3: Labeling of GST-cCPE; Method S4: Plate reader binding assay; Method S5: Preparation and GST-cCPE incubation of rat colon; Method S6: Ex vivo cCPE treatment of human colon polyps; Method S7: Histology and Immunochemistry.
