# Supplementary material for: cCPE Fusion Proteins as Molecular Probes to Detect Claudins and Tight Junction Dysregulation in Gastrointestinal Cell Lines, Tissue Explants and Patient-Derived Organoids
Source: Pharmaceutics. 2023 Jul 19;15(7):1980. doi: 10.3390/pharmaceutics15071980 (PMC10385049; doi:10.3390/pharmaceutics15071980)
Supplement: Supplementary file 1 [file pharmaceutics-15-01980-s001.zip › pharmaceutics-2435237-supplementary.pdf]

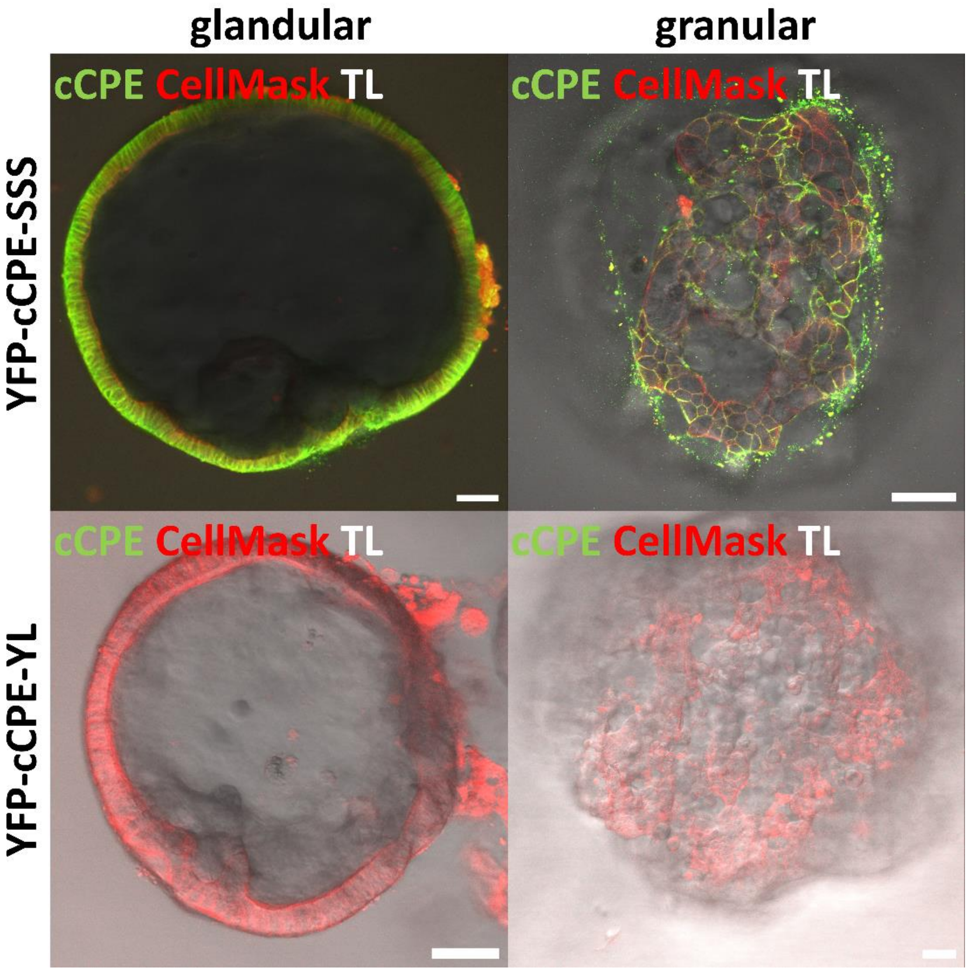

Figure S1 Live-cell imaging of gastric cancer organoids with YFP-cCPE-SSS and –YL. Glandular and granular organoids were both incubated with 15 µg/ml of YFP-cCPE-SSS or –YL and CellMask™ (1:15000) . Only YFP-cCPE-SSS gave a strong YFP signal, indicating presence of cCPE receptor claudins and specific binding in gastric cancer organoids. TL, transmitted light channel. Scale bars 50 µm.

Table S1. Culture media for cell lines and gastric organoid culture. FBS – fetal bovine serum. All media were prepared and used under sterile conditions.

Medium	Reagent	Concentration	Source	CAT#
**HT29/B6 medium**	RPMI-1640 AQMedia™	-	Thermo Fisher Scientific, Waltham, Massachusetts, USA	61870036
	Fetal Bovine Serum (FBS)	10 % (v/v)	Thermo Fisher Scientific, Waltham, Massachusetts, USA	10270-106
	penicillin	100 U/ml	Thermo Fisher Scientific, Waltham, Massachusetts, USA	15140-122
	streptomycin	100 µg/ml		
**HEK293 medium**	DMEM, high glucose, GlutaMAX™	-	Thermo Fisher Scientific, Waltham, Massachusetts, USA	D6429
	Fetal Bovine Serum (FBS)	10 % (v/v)	Thermo Fisher Scientific, Waltham, Massachusetts, USA	10270-106
	penicillin	100 U/ml	Thermo Fisher Scientific, Waltham, Massachusetts, USA	15140-122
	streptomycin	100 µg/ml		
	G418-BC	1000 U/ml	Biochrom, Berlin, Germany	A 2912
**MDCK-C7 medium**	MEM	-	Sigma-Aldrich, Taufkirchen, Germany	41090036
	Fetal Bovine Serum (FBS)	10 % (v/v)	Thermo Fisher Scientific, Waltham, Massachusetts, USA	10270-106
	penicillin	100 U/ml	Thermo Fisher Scientific, Waltham, Massachusetts, USA	15140-122
	streptomycin	100 µg/ml		
**Washing medium**	Advanced RPMI 1640 medium		Thermo Fisher Scientific, Waltham, Massachusetts, USA	12633012
	penicillin/streptomycin	100 µg/ml	Biochrom, Berlin, Germany	A2213
**Enzyme mix**	DNAse I	1.25 units/ml	Sigma-Aldrich, Taufkirchen, Germany	D4263-5VL
	hyaluronidase V	250 units/ml	Sigma-Aldrich, Taufkirchen, Germany	H6254
	dispase	0.15 units/ml	Corning, Corning, New York, USA	354235
	elastase	0.025 unit/ml	Sigma-Aldrich, Taufkirchen, Germany	E7885
	collagenase XI	0.5 mg	Sigma-Aldrich, Taufkirchen, Germany	C7657
**Organoid medium**	Advanced RPMI 1640 medium		Thermo Fisher Scientific, Waltham, Massachusetts, USA	12633012
	penicillin/streptomycin	100 µg/ml	Biochrom, Berlin, Germany	A2213
	HEPES	10 mM	Thermo Fisher Scientific, Waltham,	15630106

		Massachusetts, USA	
L-glutamine	4 mM	Thermo Fisher Scientific, Waltham, Massachusetts, USA	35050061
amphotericin b	2.5 µg/ml	Biochrom, Berlin, Germany	A2612
insulin-transferrin-selenium	5 µg/ml – 5 µg/ml – 5 ng/ml	Sigma-Aldrich, Taufkirchen, Germany	I1884
nicotinamide	10 mM	Sigma-Aldrich, Taufkirchen, Germany	72345
B-27	1%	Thermo Fisher Scientific, Waltham, Massachusetts, USA	17504044
hydrocortisone	0.48 µg/ml	Stemcell Technologies, Cologne, Germany	07925
FGF2	5 ng/ml	Stemgent, Cambridge, Massachusetts, USA	03-0002
hEGF	50 ng/ml	Sigma-Aldrich, Taufkirchen, Germany	E9644
ROCK inhibitor Y-27632 2 HCI	10 µM	Selleck Chemicals GmbH, Planegg, Germany	SEL-S1049
FGF10	200 ng/ml	Stemcell Technologies, Cologne, Germany	78037
retinoic acid	200 nM	Sigma-Aldrich, Taufkirchen, Germany	R2625
ascorbic acid	20 µg/ml	Santa Cruz, Dallas, Texas, USA	sc-394304
R-spondin-1 or<br>R-spondin-1 conditioned medium	500 ng/ml<br>50%	Thermo Fisher Scientific, Waltham, Massachusetts, USAL-WRN cells	120-38-250<br>N/A
Wnt-3a or<br>Wnt-3a conditioned medium	100 ng/ml<br>50%	R&D Systems, Minneapolis, Minnesota, USA<br>L-WRN cells	5036-WN-010<br>N/A
[Leu15]-gastrin I		Sigma-Aldrich, Taufkirchen, Germany	G9145
noggin<br><br>noggin conditioned medium	100 ng/ml<br><br>50%	Miltenyi Biotec, Bergisch Gladbach, Germany<br>or Thermo Fisher Scientific, Waltham, Massachusetts, USA<br><br>L-WRN cells	130-103-456 or 120-10C-20<br>N/A
n-acetyl-l-cysteine	1 mM	Sigma-Aldrich, Taufkirchen, Germany	A9165
A83-01	2 µM	Stemcell Technologies, Cologne, Germany	72022

Table S2. Solution and buffers

Name (chapter)	Components
Equilibration buffer (2.1.)	50 mM NaH_2PO_4, 300 mM NaCl, 20 mM imidazole, pH 7.4
Wash buffer (2.1.)	50 mM NaH_2PO_4, 300 mM NaCl, 40 mM imidazole, pH 7.4
Elution buffer (2.1.)	50 mM NaH_2PO_4, 300 mM NaCl, 300 mM imidazole, pH 7.4
modified Ringer solution (2.6.)	134.6 mM NaCl, 5.4 mM KCl, 21 mM $NaHCO_3$, 2.4 mM Na_2HPO_4, 1.2 mM $CaCl_2$, 1.2 mM $MgCl_2$, 0.6 mM NaH_2PO_4, 2 g/l glucose, 10.6 mM HEPES, 2.5 mM L-glutamine, 0.5 mM β-hydroxybutyrate, 10 mM D(+)-mannose, 50 mg/l Piperacillin, 4 mg/l Zienam
HEPES Ringer solution (2.8.1./2.9.1.)	134.6 mM NaCl, 2.4 mM Na_2HPO_4, 0.6 mM NaH_2PO_4, 5.4 mM KCl, 1.2 mM $CaCl_2$, 1 mM $MgSO_4$, 10 mM HEPES, 10 mM D-(+)-glucose
111-Ringer + Substrate (2.8.2.)	119.7 mM NaCl, 21.4 mM $NaHCO_3$, 2.5 mM Na_2HPO_4, 0.6 mM NaH_2PO_4, 5.7 mM KCl, 1.3 mM $MgCl_2$, 1.2 mM $CaCl_2$, and 10.0 mM D(+)-glucose, 4 mg/l Securopen, 4 mg/l Zienam
Blocking solution (2.9.1.)	10 % (v/v) goat serum, 1 % (w/v) BSA, 0.8 % (v/v) Triton X-100 in PBS (+Mg^{2+}/+Ca^{2+})
Buffer B (2.9.2.)	1 % (w/v) BSA, 0.05 % Triton X-100, 0.05 % Tween 20 in PBS
TRIS-EDTA-citrate (TEC) buffer (2.9.3.)	41 mM Tris, 34 mM EDTA, 23 mM $Na_3C_6H_5O_7$, pH 5.5
TRIS-buffered saline with Tween (TBS-T) (2.9.3.)	20 mM Tris/HCl, 500 mM NaCl, 0.1% Tween 20, pH 7.3
Flow buffer (2.11.)	5 mM EDTA, 1% (w/v) BSA in PBS (-Mg^{2+}/-Ca^{2+})
FACS buffer (2.12.)	0,5 % (w/v) human serum albumin in DPBS
FSDT buffer (2.1.)	20 % (v/v) methanol, 48 mM Tris-Base, 20 mM Hepes, 1 mM EDTA, 1.3 mM NaHSO3, 1.3 mM N,N-dimethyl formamide in ddH_2O
Blocking buffer Western Blot (2.1.)	1 % (w/v) PVP-40, 0.05 % (v/v) Tween 20 in ddH_2O

Table S3. Reagents and resources

Compound/Resource	Source	CAT#
**Biological samples**		
**human tissue of patients with gastric cancer**	Department of General, Visceral and Vascular Surgery, Campus Benjamin Franklin, Charité - Universitätsmedizin Berlin, Germany	N/A
**Cell culture reagents and culture dishes**		
**Washing medium, enzyme mix, organoid medium: see supplementary table 2**		
**Dulbecco's Phosphate Buffered Saline**	Thermo Fisher Scientific, Waltham, Massachusetts, USA	14200075
**Conditioned medium containing Wnt-3a, R-spondin-3 and noggin**	L-WRN cells	N/A
**12 well culture plate**	Corning, Corning, New York, USA	3512
**24 well culture plate**	Corning, Corning, New York, USA	353047
**4 well chamber slide**	Corning, Corning, New York, USA	354114
**8 well chamber slide**	Thermo Fisher Scientific, Waltham, Massachusetts, USA	155409
**Falcon™ 5 ml round bottom polystyrene test tubes, with Cell Strainer Snap Cap**	Corning, Corning, New York, USA	352235
**Cellculture flask 50 ml, 25 cm², sterile, transparent**	Greiner Bio-One GmbH, Frickenhausen, Germany	**690160**
**Additional Cell lines**		
**L-WRN (ATCC® CRL-3276™)**	ATCC, Manassas, Virginia, USA	CRL-3276
**Chemicals & reagents**		
**0.25 % Trypsin, 2.21 mM EDTA**	Corning, Corning, New York, USA	25-053-CI
**β-hydroxybutyrate**	Sigma-Aldrich, Taufkirchen, Germany	H6501-25G
**Bovine serum albumin (BSA)**	biomol, Hamburg, Germany	01400.1
**Calcium chloride ($CaCl_2$)**	Merck KGaA, Darmstadt, Germany	1.02392.1000
**D(+)-glucose**	Carl Roth, Karlsruhe, Germany	6780.1
**D(+)-mannose**	Sigma-Aldrich, Taufkirchen, Germany	63582
**DAKO block**	Agilent Technologies Inv., Santa Clara, California, USA	X0909
**DAPI**	Sigma-Aldrich, Taufkirchen, Germany	10236276001
**DMSO**	Carl Roth, Karlsruhe, Germany	A994.1
**Disodium hydrogenphosphate (Na_2HPO_4)**	Carl Roth, Karlsruhe, Germany	N350.2
**EDTA**	Merck KGaA, Darmstadt, Germany	108418
**Eosin**	Carl Roth, Karlsruhe, Germany	7089.1
**Formaldehyde**	SAV Liquid Production, Flintsbach am Inn, Germany	FN-5000-4-1

**Geltrex**	Thermo Fisher Scientific, Waltham, Massachusetts, USA	A1413202
**Glutaraldehyde**	SERVA Electrophoresis GmbH, Heidelberg, Germany	23114.01
**Glycine**	Merck KGaA, Darmstadt, Germany	1005909012
**Hematoxylin**	Merck KGaA, Darmstadt, Germany	1.09249.0500
**Histogel**	Thermo Fisher Scientific, Waltham, Massachusetts, USA	12006679
**His60 Ni Superflow Resin**	Takara Bio Inc., Kusatsu, Japan	635677
**Hoechst 33342**	Thermo Fisher Scientific, Waltham, Massachusetts, USA	H3570
**Imidazol**	Merck KGaA, Darmstadt, Germany	288-32-4
**L-glutamine**	Thermo Fisher Scientific, Waltham, Massachusetts, USA	35050061
**Magnesium chloride ($MgCl_2$)**	Carl Roth, Karlsruhe, Germany	2189.2
**Magnesium sulfate ($MgSO_4$)**	Merck KGaA, Darmstadt, Germany	105886
**Matrigel**	Corning, Corning, New York, USA	356231
**Methanol**	Avantor Performance Materials B.V., Radnor, Pennsylvania, USA	9863-01
**Paraformaldehyde**	Electron Microscopy Sciences, Hatfield, Pennsylvania, USA or Science Service GmbH, München, Germany	15710 or E15700
**Periodic acid**	Carl Roth, Karlsruhe, Germany	3257.1
**Piperacillin**	LKT Laboratories, Minneapolis, Minnesota, USA	P3462
**Polyvinylidene difluoride membrane (PolyScreen)**	PerkinElmer, Waltham, Massachusetts, USA	NEF1002
**Potassium chloride (KCl)**	Carl Roth, Karlsruhe, Germany	P017.3
**ProTaqs MountFluor**	quartett, Berlin, Germany	401603392
**PVP-40**	Sigma-Aldrich, Taufkirchen, Germany	PVP40
**Schiff's reagent**	Sigma-Aldrich, Taufkirchen, Germany	109033
**Securopen**	Bayer, Leverkusen, Germany	N/A
**Simethicone (Sab Simplex®)**	Pfizer Consumer Healthcare, Berlin Germany	N/A
**Sodium chloride (NaCl)**	Carl Roth, Karlsruhe, Germany	3957.2
**Sodium citrate ($Na_3C_6H_5O_7$)**	Sigma-Aldrich, Taufkirchen, Germany	S4641
**Sodium dihydrogenphosphate (NaH_2PO_4)**	Carl Roth, Karlsruhe, Germany	N350.1
**Sodium hydrogencarbonate ($NaHCO_3$)**	Carl Roth, Karlsruhe, Germany	HN01.2
**Sodium hydrogensulfate ($NaHSO_4$)**	Merck KGaA, Darmstadt, Germany	106352
**Tris/Base**	Carl Roth, Karlsruhe, Germany	4855.1
**Tris/HCl**	Carl Roth, Karlsruhe, Germany	9090.3

**Triton X-100**	SERVA Electrophoresis GmbH, Heidelberg, Germany	3979501
**TrypLE Express**	Thermo Fisher Scientific, Waltham, Massachusetts, USA	12604021
**Tween 20**	Thermo Fisher Scientific, Waltham, Massachusetts, USA	233360010
**Zienam**	MSD Sharp & Dohme GmbH, Berlin, Germany	4697.01.00
**Kits**		
**Alexa Fluor®647 Protein Labeling Kit**	Molecular Probes Inc., Eugene, Oregon, USA	A20173
***Quick*-DNA Miniprep Plus Kit**	Zymo Research, Freiburg, Germany	D4068
**PAS staining kit**	Carl Roth, Karlsruhe, Germany	HP01.1
**Pierce™ BCA Protein Assay Kit**	Thermo Fisher Scientific, Waltham, Massachusetts, USA	23225
**Pierce™ ECL Plus Western Blotting Substrate**	Thermo Fisher Scientific, Waltham, Massachusetts, USA	32132
**Oligonucleotides**		
**Primer cCPE-SalI forward (AAAGTCGACGATATAGAAAAAGAAATCCTTGATTTAGCTGC)**	Eurofins Genomics, Ebersberg, Germany	N/A
**Primer cCPE-SSS-NotI reverse (AAAGCGGCCGCTTAAAATTTTTGAAATAATATGTGATAAGG)**	Eurofins Genomics, Ebersberg, Germany	N/A
**Primer cCPE-YALA-NotI reverse (AAAGCGGCCGCTTAAAATTTTTGAAATGCTATTGAATAAGG)**	Eurofins Genomics, Ebersberg, Germany	N/A
**Ion AmpliSeq Cancer Hotspot panel v2**	Thermo Fisher Scientific, Waltham, Massachusetts, USA	4475346
**Plasmids**		
**pET-28a-eYFP-cCPEwt**	Leibniz-Forschungsinstitut für Molekulare Pharmakologie, Berlin	N/A
**pGEX-4T1- GST-CPE194–309**		N/A
**pGEX-4T1-cCPE-Y3306A/L315A**		N/A
**pGEX-4T1-cCPE-S305P/S307R/S313H**		N/A
**pET-28a**	Novagen, Merck KGaA, Darmstadt, Germany	69864
**pEYFP-N1**	Clontech/ Takara Bio Inc., Kusatsu, Japan	6006-1
**Proteins**		
**cCPE-S305P/S307R/S313H**		N/A
**cCPE-Y306A/L315A**		N/A
**Equipment**		
**Inverted light microscope**	Primovert with Axiocam 105 color camera, Zeiss, Oberkochen, Germany<br>Eclipse TS 100, Nikon, Tokio, Japan	N/A
**Laser scanning microscope**	LSM 510 META, Zeiss, Oberkochen, Germany<br>LSM 780, Zeiss, Oberkochen,	N/A

	Germany	
**Objective**	LD C-Apochromat 40×/1.1 water immersion objective Zeiss, Oberkochen, Germany	N/A
**Imaging software**	ZEN lite, Zeiss, Oberkochen, Germany<br>ZEN Black, Zeiss, Oberkochen, Germany	N/A
**Trans-Blot Turbo Transfer System**	Bio-Rad Laboratories Inc., Hercules, California, USA	N/A
**Plate Reader**	Infinite® M200, Tecan Trading AG, Männedorf, Switzerland	N/A
**Chemiluminescence imaging system**	Fusion FX7, Vilber Lourmat, Eberhardzell, Germany	N/A
**Flow Cytometer**	BD LSRFortessa X-20, BD BioSciences, Franklin Lakes, New Jersey, USA<br>CytoFlex, Beckman coulter Life Sciences, Indianapolis, Indiana, USA	N/A
**Next-generation sequencing (NGS) system**	Ion GeneStudio™ S5, Thermo Fisher Scientific, Waltham, Massachusetts, USA	N/A
**Software**		
**Zen lite**	Zeiss, Oberkochen, Germany	N/A
**Zen Black**	Zeiss, Oberkochen, Germany	N/A
**BD FACSDiva™**	BD Biosciences, Franklin Lakes, New Jersey, USA	N/A
**Floreada**	Floreada.io	N/A
**FlowJo**	Version 10.8.2, BD Biosciences, Franklin Lakes, New Jersey, USA	N/A
**Fiji**	1.53t, [1]	N/A

Table S4. Immunofluorescence staining protocol for HT-29/B6 cells and whole mount samples of rat colon and colon polyp biopsies. Unless otherwise stated, all steps were performed at room temperature. 60 µl of respective solutions were used for 12 mm coverslips. Volumina for processing of whole mount samples was adjusted depending on tissue size. Samples were covered by solutions at all times. See also Method S7 Histology and Immunochemistry bellow.

Procedure	Cells		Whole mount samples	
	**Solution**	**Incubation period**	**Solution**	**Incubation period**
Fixation	2% (w/v) PFA	20 min	2% (w/v) PFA	2 h
Quenching	125 mM glycine	5 min	125 mM glycine	Over night, 4 °C
Permeabilization	0.5% (v/v) Triton X-100	5 min	1% (v/v) Triton X-100	2 h
Washing	PBS	2 × 1 min	-	
Blocking	Buffer B	10 min	Blocking solution	3 h
Primary antibody 1	Buffer B	1 h	Blocking solution	Over night, 4 °C
washing	Buffer B	1 min	Blocking solution	1 × 1 min<br>1 × 10 min<br>2 × 1 h
Primary antibody 2	Buffer B	1 h	Blocking solution	Over night, 4 °C
washing	Buffer B	5 × 2 min	Blocking solution	1 × 1 min<br>1 × 10 min<br>2 × 1 h
Secondary antibody	Buffer B + DAPI (1:500)	30 min	Blocking solution	Over night, 4 °C
washing	Buffer B	5 × 2 min	Blocking solution	1 × 1 min<br>1 × 10 min<br>2 × 1 h
staining			Blocking solution with DAPI (1:500)	1 h
washing			PBS<br>ddH_2O	2 × 15 min<br>1 × 15 min

Table S5. Primary and secondary antibodies and fluorophores. Dilutions were prepared in blocking buffers. ex – excitation wavelength, em – emission wavelength. Wavelength are given as range that was used for excitation or detection at the LSM.

Primary antibody	Fluorophore	dilution	manufacturer	species	clone	Catalogue number
anti-GST	-	1:100	Sigma-Aldrich, Taufkirchen, Germany	mouse	6G9C6	SAB5300159
anti- ZO-1	-	1:100	Invitrogen, Waltham, Massachusetts, USA	rabbit	Polyclonal	61-7300
anti-Clnd1	-	1:100	Invitrogen, Waltham, Massachusetts, USA	rabbit	Polyclonal	51-9000
anti-Cldn3	-	1:100	Invitrogen, Waltham, Massachusetts, USA	rabbit	Polyclonal	34-1700
anti-Cldn4	-	1:100	Invitrogen, Waltham, Massachusetts, USA	rabbit	Polyclonal	PA5-16875
anti-GFP	-	1:100	Clontech Laboratories Inc.	mouse	JL-8	632381
anti-e-cadherin	AlexaFluor™ 647	1:100	BD Biosciences, Franklin Lakes, New Jersey, USA	mouse	67A4	560062
anti-CEA	-	1:100	Cell Signaling, Danvers, Massachusetts, USA	mouse	CB30	42738
anti-CK19	-	1:100	Thermo Fisher Scientific, Waltham, Massachusetts, USA	mouse	RCK108	MA5-12613
anti-vimentin	-	1:200	abcam, Cambridge, UK	rabbit	EPR3776	ab92547?

anti- PDL1	-	1:200	Cell Signaling, Danvers, Massachusetts, USA	rabbit	E1L3N	13684
anti-TP53	-	1:50	Agilent Technologies Inv., Santa Clara, California, USA	mouse	DO-7	M 7001
anti-PDGFRα	BV421	1:200	BD Biosciences, Franklin Lakes, New Jersey, USA	mouse	aR1	562799
anti-CD44	APC	1:200	BioLegend, San Diego, California, USA	rat	C44 Mab-5	103012
anti-CD166	APC F750	1:400	BioLegend, San Diego, California, USA	mouse	3A6	343910
anti-CD45	FITC	1:400	BioLegend, San Diego, California, USA	mouse	HI30	304006
anti-EpCAM	PE	1:100	BioLegend, San Diego, California, USA	mouse	9C4	324206
anti-CD31	PerCpCy5.5	1:400	BioLegend, San Diego, California, USA	mouse	WM59	303132
anti-CD133	PeCy7	1:400	BioLegend, San Diego, California, USA	mouse	clone 7	372810
**Secondary antibody**						
anti-mouse	Alexa Fluor™ Plus 488	1:250	Invitrogen, Waltham, Massachusetts, USA	goat	Polyclonal	A32723
anti-mouse	Alexa Fluor™ 549	1:250	Invitrogen, Waltham, Massachusetts, USA	goat	Polyclonal	A-11032
anti-rabbit	Alexa Fluor™ 488	1:250	Invitrogen, Waltham, Massachusetts, USA	goat	Polyclonal	A-11034
anti-rabbit	Alexa Fluor™ Plus 594	1:250	Invitrogen, Waltham, Massachusetts, USA	goat	Polyclonal	A32740
anti-mouse HRP	-	1:10 000	Jackson Immuno Research Laboratories Inc.	goat	Polyclonal	115-035-003
**Other Dyes**						
CellMask™ Deep Red Plasma membrane stain	N/A	1:15 000	Thermo Fisher Scientific, Waltham, Massachusetts, USA	-	-	C10046
CellMask™ Orange plasma membrane stain	N/A	1:1000 (FACS)	Thermo Fisher Scientific, Waltham, Massachusetts, USA			C10045
LIVE/DEAD™ Fixable Violet Dead Cell Stain	N/A	1:10 000	Thermo Fisher Scientific, Waltham, Massachusetts, USA	-	-	L23105
Chromotek GFP-booster ATTO647N	ATTO647N	1:100 (IF), 1:500 (FACS)	Chromotek, Planegg, Germany	alp	-	gba647n

Supplementary Methods

Method S1 Plasmids

Plasmids encoding fusion proteins of GST and the carboxy-terminal domain, i.e. residues 194 – 319, of CPE (henceforth referred to as GST-cCPE) and variants thereof were described previously [2].

Plasmid pET-28a-eYFP-cCPEwt encoding a fusion protein of 6 × His tag, eYFP and cCPEwt (from N- to C-terminus) was generated by PCR

and ligation-based subcloning of eYFP from pEYFP-N1 using BamHI and SacI restriction sites and cCPEwt from pGEX-4T1- GST-CPE194–309 using SalI and NotI restriction sites into pET-28a. This plasmid was further used for site-directed mutagenesis. A SalI restriction site at the 5`-end and a NotI restriction site at the 3`-end of the cCPE sequences were introduced using the plasmids pGEX-4T1-cCPE-Y306A/L315A and pGEX-4T1-cCPE-S305P/S307R/S313H as templates for mutagenesis PCR [2]. The primers used were as follows: cCPE-SalI forward, cCPE-YALA-NotI reverse, and cCPE-SSS-NotI reverse. The PCR fragments and the pET-28a-eYFP-cCPEwt construct were digested with SalI and NotI restriction enzymes and the digested fragments of cCPE-YALA and cCPE-SSS were ligated into the cut pET-28a-eYFP-cCPEwt plasmid, yielding plasmids pET-28a-eYFP-cCPE-Y306A/L315A and pET-28a-eYFP-cCPE-S305P/S307R/S313H.

Method S2 Purification of 6×His-eYFP-cCPE proteins

The 6 × His-eYFP-cCPE proteins were expressed in Escherichia coli BL21 (DE3, generated as described in [3]) and purified from lysates as described earlier [4]. Buffers for protein purification were prepared according to the His60 Ni Superflow™ Resin & Gravity Column User Manual. In short, cleared lysates were loaded onto columns containing Ni-IDA-Agarose, washed with equilibration and wash buffer, followed by elution of proteins using a high concentration of imidazole in the elution buffer. EYFP fluorescence from the eluates was analyzed in a plate reader and purity of the samples was assessed via SDS-PAGE with subsequent Coomassie staining. Eluates having high purity and eYFP fluorescence were dialyzed against DPBS and the concentration of the proteins was determined by a BCA protein assay.

Identity of the purified protein was confirmed by Western Blotting using mouse anti-GFP/YFP antibody.

Method S3 Labeling of GST-cCPE

GST-cCPE variants were labeled with Alexa Fluor®647 using Alexa Fluor®647 Protein Labeling Kit. The protein labeling kit uses an amino-reactive fluorophore, which forms covalent bonds with accessible lysine (K) residues of the target protein. As cCPE residue K257 is located in close proximity to the claudin binding pocket, variant GST-cCPE-K257A was used instead of GST-cCPEwt. This mutant exhibits claudin binding properties similar to the wild type protein, and was used to prevent steric hindrance of claudin binding due to the conjugated fluorophore.

Method S4 Plate reader binding assay

Prior to performing binding assays, HEK293-CLDN4 cells were seeded at 60 % confluency in 24 well plates and grown over night at 37 °C and 5 % CO_2. Protein concentration of YFP-cCPE fusion proteins were measured by using the Pierce™ BCA protein assay kit. HEK293-CLDN4 were incubated with increasing concentrations of YFP-cCPE fusion proteins (0 – 10 µg/ml, 37 °C, 5 % CO_2, 30 min) and Hoechst 33342 (2 mM). Cells were then washed with ice-cold PBS (+Mg^{2+}/+Ca^{2+}) and fluorescence intensity was measured. YFP signal was normalized to Hoechst 33342 signal, reflecting cell number. Significance was tested with two-way ANOVA to compare concentration and cCPE variants, with post-hoc Turkey test to account for multiple comparison. Significance thresholds are given in the respective figures.

Method S5 Preparation and GST-cCPE incubation of rat colon

Male Wistar rats (m = 250 – 300 g) were housed under standardized conditions (12-hour light/dark cycle; 22 – 24 °C temperature; 55 % ± 15 % humidity; ad libitum access to standard diet and water) (according to the EU guidelines 010/63/EU, 2007/526/EG and GV-SOLAS), and euthanized by CO_2 inhalation, followed by bilateral thoracotomy (in accordance with the German law on animal protection (Landesamt für Gesundheit und Soziales, Berlin T0255/05). The colon (15 cm from rectum) was harvested, rinsed with modified Ringer solution, opened longitudinally and the tunica serosa, muscularis propria and tunica submucosa were removed [5]: On a plastic plate, the tissue was placed with the mucosal side down and the muscularis propria was removed using fine forceps. Afterwards, the tissue was turned (mucosal side facing up) and the mucosa (lamina epithelialis mucosae, lamina propria mucosae, lamina muscularis mucosae) peeled off manually and used for further experiments. Tissue pieces were mounted into custom made (Charité – Universitätsmedizin Berlin) Ussing chambers [6]. By these means, 0.54 cm^2 of colon epithelium exposed per Ussing chamber [7,8], with one colon yielding 6 – 8 pieces of tissue samples.

10 ml of carbogen-flushed modified Ringer solution with 10 % (v/v) heat-inactivated fetal bovine serum and 0.5 % (v/v) simethicone to prevent foaming were gently added to both sides of the Ussing chambers. After equilibration for 30 min at 37 °C and under constant carbogen flushing, transepithelial resistance was measured to assure tissue integrity. Short-circuit current, open-circuit transepithelial voltage, and transepithelial resistance of the tissues were recorded. The transepithelial resistance of intact biopsies ranged from 50 to 160 $\Omega \cdot cm^2$ [5]. The samples were incubated for 20 h [5]. Subsequently, pre-warmed modified Ringer solution supplied with 10 µg/ml of GST-cCPE-SSS or –YL was added to the serosal side and incubated for 1 h at 37 °C. The tissue was carefully removed from the Ussing chambers, fixed with 2 % PFA (prepared from 16 % (w/v) aqueous solution) for 2 h at room temperature, and quenched in 125 mM glycine overnight at 4 °C. Further processing as whole mount samples was performed as described under *Methods S7.3 IF staining of whole mount ex vivo model systems.*

Method S6 Ex vivo cCPE treatment of human colon polyps

Biopsies of human colon polyps were obtained from otherwise healthy colon mucosa during routine endoscopy. Immediately following polypectomy, tissue biopsies were placed in 0.9 % (w/v) NaCl solution on ice. Further processing took place within 30 min. If necessary, biopsies were cut into smaller pieces and placed in pre-heated, carbogen-flushed ringer solution containing either 15 µg/ml GST-cCPE-SSS or -YL, respectively. Tissue was incubated in GST-cCPE solution for 30 min at 37 °C and inverted regularly. Subsequently, samples were fixed in 2 % (w/v) PFA for 2 h at room temperature, and quenched in 125 mM glycine over night at 4 °C. Further processing as whole mount samples was performed as described under *Method S7.3 IF staining of whole mount ex vivo model systems.*

Method S7 Histology and Immunochemistry

Method S7.1 Paraffin sectioning, hematoxylin, eosin and periodic acid-Schiff staining of primary gastric cancer tissue and patient-derived organoids

After fixation for 24 h in 4 % formaldehyde, primary tissue samples were embedded in paraffin and cut into 4 µm sections. Direct embedding of organoids in Histogel was performed as described previously [9]. Briefly, the culture medium was removed and the wells were washed with DPBS. Matrigel / Geltrex-embedded organoids were transferred into a cryomold with a solidified Histogel base. Liquid Histogel (heated at 65 °C) was added on top of the Matrigel and cooled for at least 1 h on ice. The Histogel block was transferred into a tissue cassette and placed in a 4 % formaldehyde solution overnight or 4 % PFA / 0.5 % glutaraldehyde for 2 h at 25 °C. Then the cassette was stored in 70 % ethanol until paraffin embedding. Organoid processing in Histogel blocks was performed as described previously [10].

Paraffin sections of the primary tissue and Histogel-embedded organoids were deparaffinized using a standardized protocol. Sections were stained with hematoxylin and eosin, 1 % periodic acid and Schiff´s reagent.

Method S7.2 Immunofluorescence (IF) staining of subconfluent HT-29/B6

Procedures are described in detail in **Table** S4. Briefly: HT-29/B6 cells were fixed in 2 % PFA, quenched with 125 mM glycine, permeabilized with 0.5 % Triton X-100 and blocked in buffer B. Primary and secondary antibody incubation each were done for 1 h at room temperature. Samples were stained for rabbit anti-Cldn3 (1:100) and mouse anti-GST (1:100) was used to detect GST-cCPE. For secondary antibodies anti-rabbit AlexaFluor 594 (1:250) and anti-mouse AlexaFluor 488 (1:250) were used. GST-cCPE incubations were done before fixation in HEPES Ringer solution at 10 µg/ml of respective GST-cCPE fusion protein for 30 min at 37 °C. Confocal images were acquired with a LSM780. Images were processed using Zen Black and Fiji.

Method S7.3 IF staining of whole mount ex vivo model systems

Details can be found in **Table** S4. In brief, quenched rat colon and human colon polyp samples were permeabilized with 1 % Triton X-100 followed by incubation in blocking solution. Primary and secondary antibodies were applied over night at 4 °C. cCPE was detected either via coupled AF647 or via primary mouse anti-GST antibody (1:100), while ZO1 was detected by rabbit anti-ZO1 (1:100). For secondary antibodies anti-mouse Alexa Fluor 594 (1:250) and anti-rabbit Alexa Fluor 488 (1:250) were used, and nuclei were stained with DAPI (1:1000).

Method S7.4 IF staining of gastric tissue and organoid sections

Paraffin sections of the primary tissue and organoids were rehydrated with xylene for 20 min three times and incubated in an ethanol dilution series and distilled water.

Antigen retrieval was done by boiling sections in TEC buffer for 30 min. Once cooled to room temperature sections were washed twice in TBS-T, incubated with DAKO Antibody Diluent with Background Reducing Components for 30 min at 25 °C and incubation with antibodies against Cldn1, Cldn4 and GFP was performed at 4 °C over night. After washing with TBS-T, paraffin sections were incubated with secondary antibodies (goat anti-mouse Alexa Fluor®Plus 488, goat anti-rabbit Alexa Fluor®Plus 594) and DAPI (1:1000) for 90 min at 37 °C.

Sections were then washed in TBS-T, followed by incubation with primary E-cadherin antibody directly coupled with Alexa Fluor®647. Sections were rinsed with TBS-T and distilled water before embedding in Mount Fluor (ProTaqs).

IF staining's for tumor markers on paraffin sections followed an alternative previously described protocol [11]. This protocol was conducted for staining of CEA, CK19 and vimentin shown in **Figure** 4.

Method S7.5 Immunohistochemistry gastric tissue / organoids

For immunohistochemistry stainings, sections were incubated in CC1 mild buffer (Ventana Medical Systems) for 30 min at 100 °C or in protease 1 for 8 min. Afterwards, the sections were stained against PD-L1 (programmed death ligand 1, 1:200) or p53 (1:50) for 60 min at room temperature and visualized using the avidin-biotin complex method and DAB. For this purpose, the BenchMark XT immunostainer (Ventana Medical Systems, Tucson, Arizona, United States) was used. The counterstaining of the cell nuclei was done by incubating for 12 min with hematoxylin and bluing reagent (Ventana Medical Systems, Tucson, Arizona, United States). Slides were assessed using Primovert (Zeiss, Oberkochen, Germany) microscope with Axiocam 105 color camera (Zeiss, Oberkochen, Germany) and ZEN imaging software.

References

1. Schindelin, J.; Arganda-Carreras, I.; Frise, E.; Kaynig, V.; Longair, M.; Pietzsch, T.; Preibisch, S.; Rueden, C.; Saalfeld, S.; Schmid, B.; et al. Fiji: an open-source platform for biological-image analysis. *Nat Methods* **2012**, *9*, 676-682, doi:10.1038/nmeth.2019.
2. Protze, J.; Eichner, M.; Piontek, A.; Dinter, S.; Rossa, J.; Blecharz, K.G.; Vajkoczy, P.; Piontek, J.; Krause, G. Directed structural modification of Clostridium perfringens enterotoxin to enhance binding to claudin-5. *Cellular and molecular life sciences : CMLS* **2015**, *72*, 1417-1432, doi:10.1007/s00018-014-1761-6.
3. Studier, F.W.; Rosenberg, A.H.; Dunn, J.J.; Dubendorff, J.W. Use of T7 RNA polymerase to direct expression of cloned genes. *Methods in enzymology* **1990**, *185*, 60-89, doi:10.1016/0076-6879(90)85008-c.
4. Eichner, M.; Augustin, C.; Fromm, A.; Piontek, A.; Walther, W.; Bucker, R.; Fromm, M.; Krause, G.; Schulzke, J.D.; Gunzel, D.; et al. In Colon Epithelia, Clostridium perfringens Enterotoxin Causes Focal Leaks by Targeting Claudins Which are Apically Accessible Due to Tight Junction Derangement. *J Infect Dis* **2017**, *217*, 147-157, doi:10.1093/infdis/jix485.
5. Amasheh, M.; Grotjohann, I.; Amasheh, S.; Fromm, A.; Soderholm, J.D.; Zeitz, M.; Fromm, M.; Schulzke, J.D. Regulation of mucosal structure and barrier function in rat colon exposed to tumor necrosis factor alpha and interferon gamma in vitro: a novel model for studying the pathomechanisms of inflammatory bowel disease cytokines. *Scand J Gastroenterol* **2009**, *44*, 1226-1235, doi:10.1080/00365520903131973.
6. Kreusel, K.M.; Fromm, M.; Schulzke, J.D.; Hegel, U. Cl- secretion in epithelial monolayers of mucus-forming human colon cells (HT-29/B6). *Am J Physiol* **1991**, *261*, C574-582, doi:10.1152/ajpcell.1991.261.4.C574.
7. Epple, H.J.; Amasheh, S.; Mankertz, J.; Goltz, M.; Schulzke, J.D.; Fromm, M. Early aldosterone effect in distal colon by transcriptional regulation of ENaC subunits. *Am J Physiol Gastrointest Liver Physiol* **2000**, *278*, G718-724, doi:10.1152/ajpgi.2000.278.5.G718.
8. Fromm, M.; Schulzke, J.D.; Hegel, U. Control of electrogenic Na+ absorption in rat late distal colon by nanomolar aldosterone added in vitro. *Am J Physiol* **1993**, *264*, E68-73, doi:10.1152/ajpendo.1993.264.1.E68.

9. Pinto, M.P.; Jacobsen, B.M.; Horwitz, K.B. An immunohistochemical method to study breast cancer cell subpopulations and their growth regulation by hormones in three-dimensional cultures. *Front Endocrinol (Lausanne)* **2011**, *2*, 15, doi:10.3389/fendo.2011.00015.
10. Huang, L.; Holtzinger, A.; Jagan, I.; BeGora, M.; Lohse, I.; Ngai, N.; Nostro, C.; Wang, R.; Muthuswamy, L.B.; Crawford, H.C.; et al. Ductal pancreatic cancer modeling and drug screening using human pluripotent stem cell- and patient-derived tumor organoids. *Nat Med* **2015**, *21*, 1364-1371, doi:10.1038/nm.3973.
11. Ehlen, L.; Arndt, J.; Treue, D.; Bischoff, P.; Loch, F.N.; Hahn, E.M.; Kotsch, K.; Klauschen, F.; Beyer, K.; Margonis, G.A.; et al. Novel methods for in vitro modeling of pancreatic cancer reveal important aspects for successful primary cell culture. *BMC Cancer* **2020**, *20*, 417, doi:10.1186/s12885-020-06929-8.
